# Prostate Tumor Overexpressed-1 (PTOV1) promotes docetaxel-resistance and survival of castration resistant prostate cancer cells

**DOI:** 10.18632/oncotarget.19467

**Published:** 2017-07-22

**Authors:** Verónica Cánovas, Yolanda Puñal, Valentina Maggio, Enric Redondo, Mercedes Marín, Begoña Mellado, Mireia Olivan, Matilde Lleonart, Jacques Planas, Juan Morote, Rosanna Paciucci

**Affiliations:** ^1^ Biomedical Research Group of Urology, Vall d’Hebron Research Institute, Universitat Autònoma de Barcelona, Barcelona, Spain; ^2^ Laboratory of Translational Genomics and Targeted Therapeutics in Solid Tumors, August Pi i Sunyer Biomedical Research Institute (IDIBAPS), Medical Oncoloy Department, Hospital Clinic, Barcelona, Spain; ^3^ Biomedical Research in Cancer Stem Cells, Vall d’Hebron Research Institute, Universitat Autònoma de Barcelona, Barcelona, Spain; ^4^ Deparment of Urology, Vall d’Hebron Hospital, Universitat Autònoma de Barcelona, Barcelona, Spain

**Keywords:** PTOV1, docetaxel resistance, prostate cancer, metastasis, apoptosis

## Abstract

Metastatic prostate cancer is presently incurable. The oncogenic protein PTOV1, first described in prostate cancer, was reported as overexpressed and significantly correlated with poor survival in numerous tumors. Here, we investigated the role of PTOV1 in prostate cancer survival to docetaxel and self-renewal ability. Transduction of PTOV1 in docetaxel-sensitive Du145 and PC3 cells significantly increased cell survival after docetaxel exposure and induced docetaxel-resistance genes expression (*ABCB1, CCNG2* and *TUBB2B*). In addition, PTOV1 induced prostatospheres formation and self-renewal genes expression (*ALDH1A1, LIN28A, MYC* and *NANOG*). In contrast, Du145 and PC3 cells knockdown for PTOV1 significantly accumulated in the G2/M phase, presented a concomitant increased subG1 peak, and cell death by apoptosis. These effects were enhanced in docetaxel-resistant cells. Analyses of tumor datasets show that *PTOV1* expression significantly correlated with prostate tumor grade, drug resistance (*CCNG2*) and self-renewal (*ALDH1A1, MYC*) markers. These genes are concurrently overexpressed in most metastatic lesions. Metastases also show PTOV1 genomic amplification in significant co-occurrence with docetaxel-resistance and self-renewal genes. Our findings identify PTOV1 as a promoter of docetaxel-resistance and self-renewal characteristics for castration resistant prostate cancer. The concomitant increased expression of *PTOV1*, *ALDH1A1* and *CCNG2* in primary tumors, may predict metastasis and bad prognosis.

## INTRODUCTION

Prostate cancer is the most common male malignancy and the second leading cause of death for cancer in men of western Countries [1]. The majority of newly diagnosed localized tumors are successfully treated by surgery. Patients with metastatic prostate cancer undergo androgen deprivation therapy (ADT), very effective in reducing prostate tumor and metastatic growth. However, progression to a castration-resistant prostate cancer (CRPC) eventually occurs [2, 3]. Docetaxel, a taxol derived drug that prevents microtubule de-polymerization and mitotic division, was the standard treatment in US for CRPC patients until 2010 [4]. More recently, docetaxel combined with androgen deprivation therapy have been used at earlier stages to improve the outcome for men with metastases at first presentation [5–7], but unfortunately in most cases it results in the development of a very aggressive resistant cancer.

PTOV1 was previously demonstrated to be pro-oncogenic in prostate cancer [8–11]. The protein is strongly expressed in primary tumors and metastatic lesions [9–11]. Its detection in premalignant lesions of high grade prostate intraepithelial neoplasia (HGPIN) is helpful to identify patients with significantly higher probability to develop prostate cancer [12, 13]. PTOV1 is overexpressed and significantly associated to a high grade of malignancy, increased proliferation, and unfavorable prognosis in a number of other neoplasias [13–18]. Its expression induces spheroid formation *in vitro*, tumor growth, invasion and metastasis of CRPC cells [10, 11]. Mechanistic studies have shown that PTOV1 cytoplasmic localization is associated to activation of translation of a subset of mRNAs through the interaction with RACK1 and ribosomes, leading to a specific increased synthesis of the c-Jun oncogene [10]. In turn, c-Jun was shown to promote *SNAI1* transcription and the activation of an *epithelial-mesenchymal transition* (EMT) program, leading to tumor growth and metastasis [10]. In the nucleus, PTOV1 acts as a transcriptional repressor of *HES1* and *HEY1* genes, two main targets of the Notch receptor, thus revealing its oncogenic function in counteracting the tumor suppressor action of Notch signaling in advanced prostate cancer [11]. PTOV1 is a transcriptional repressor of the *DKK1* gene, an action that triggers the activation of the Wnt/β-catenin signaling and tumor progression in breast cancer cells [16], and of the retinoic acid receptor (*RARβ*), favoring cell resistance to retinoic acid [19]. A recent review about the known oncogenic mechanisms of PTOV1 in tumors is available [20].

The resistance to docetaxel in treated tumors is associated to the expression of alternative β-tubulin isoforms, genes encoding for drug efflux pumps, and survival factors [21–23]. In addition, resistant metastatic growth has been associated to rare populations of slow growing cells within tumors identified as cancer stem cells (CSCs) that have intrinsic resistance to therapeutic stress [24–26]. Recent data suggest that taxanes can also affect androgen receptor (AR) signaling by inhibiting ligand-induced AR nuclear translocation and downstream transcriptional activation of its target genes and confirming a role for microtubules in AR trafficking [27]. However, AR nuclear translocation and signaling was not inhibited in cells with acquired β-tubulin mutations, that prevent taxanes-induced microtubule stabilization [28]. In addition, c-Jun, SNAI1, and NOTCH2/Hedgehog pathways have been implicated in the development of resistance to docetaxel or paclitaxel [23, 29, 30]. However, despite the intense research, the mechanisms of prostate cancer resistance to docetaxel are not completely understood and more investigations are required to design targeted therapies able to overcome its insurgence.

Here, our findings reveal a critical function for PTOV1 in the activation of genes implicated in the acquisition of resistance to docetaxel and the appearance of a stemness-*like* phenotype. These results are confirmed by analyses of datasets from human prostate tumors and reveal a specific and significant direct correlation of *PTOV1* with *ALDH1A1* and *CCNG2*. These findings identify PTOV1 as a novel potential therapeutic target for patients with metastatic prostate cancer.

## RESULTS

### PTOV1 promotes docetaxel resistance in CRPC cells

**Figure 1:**
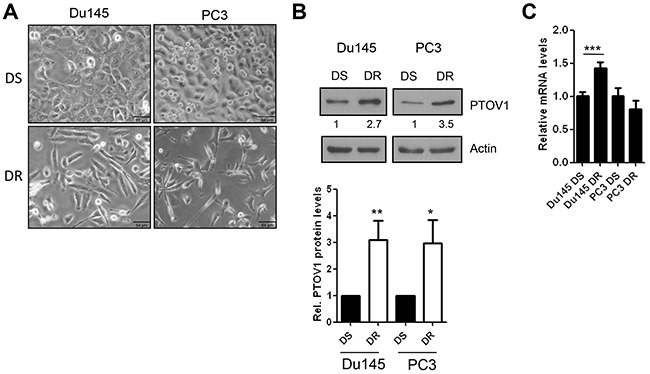
PTOV1 is overexpressed in docetaxel resistant CRPC cell lines **(A)** Phase contrast images of docetaxel sensitive (DS) and resistant (DR) Du145 and PC3 cells in culture. Size bar, 64 μm. Images were acquired with an inverted microscope (BX61, Olympus). **(B)** A representative immunoblot shows the expression of endogenous PTOV1 in Du145 and PC3 cells. The graph below shows the average of expression of PTOV1 from three independent immunoblots, two of which are shown in Supplementary Figure 1B. **(C)** Endogenous mRNA levels of PTOV1 (mean ± S.D.) determined by real-time RT-PCR.

Increased mRNA and protein levels of PTOV1 were shown to correlate with prostate cancer progression [10, 11] and to promote CSCs-*like* properties [11, 16]. Here, we investigated whether its overexpression in prostate cancer cells is associated to the acquisition of resistance to a therapeutic stress. Thus, PTOV1 expression was analyzed in Du145 and PC3 prostate cancer cells rendered resistant to docetaxel *in vitro* as representative models of CRPC progression to a docetaxel resistant (DR) stage [31]. DR-Du145 and DR-PC3 cells show an evident mesenchymal phenotype (Figure 1A), as previously described [31, 32], a very significant decrease in epithelial markers, and overexpression of genes implicated in the acquisition of drug resistance, previously reported in taxanes resistant cells [31–36]. In contrast to its low levels in benign prostate derived RWPE1 cells, PTOV1 is strongly expressed in most prostate carcinoma cell lines (Supplementary Figure 1A). Both DR-Du145 and DR-PC3 cell variants have a consistently increased protein levels for PTOV1 compared with parental docetaxel sensitive (DS) cells (Figure 1B and Supplementary Figure 1B), and a significant increase in RNA levels is observed in DR-Du145 but not in DR-PC3 cells (Figure 1C). To address whether translation rates may contribute to increase PTOV1 protein levels in DR cells, we analyzed the levels of PTOV1 transcripts more actively translated by studying the amount of mRNA loaded on polysomes (Supplementary Figure 2A). No significant differences are found comparing the total (DR-T) and polysomes-associated mRNA levels (DR-P) in DR cells compared to control DS cells, suggesting that the higher protein expression observed in DR cells is not contributed by an enhanced protein synthesis. In addition, although a significant increase in PTOV1 protein stability is detected in cycloheximide (CHX)-treated DR-Du145 cells, no significant differences were detected in DR-PC3 cells, suggesting that the mechanisms underlying the higher PTOV1 protein expression in DR cells need to be explored further (Supplementary Figure 2B).

**Figure 2:**
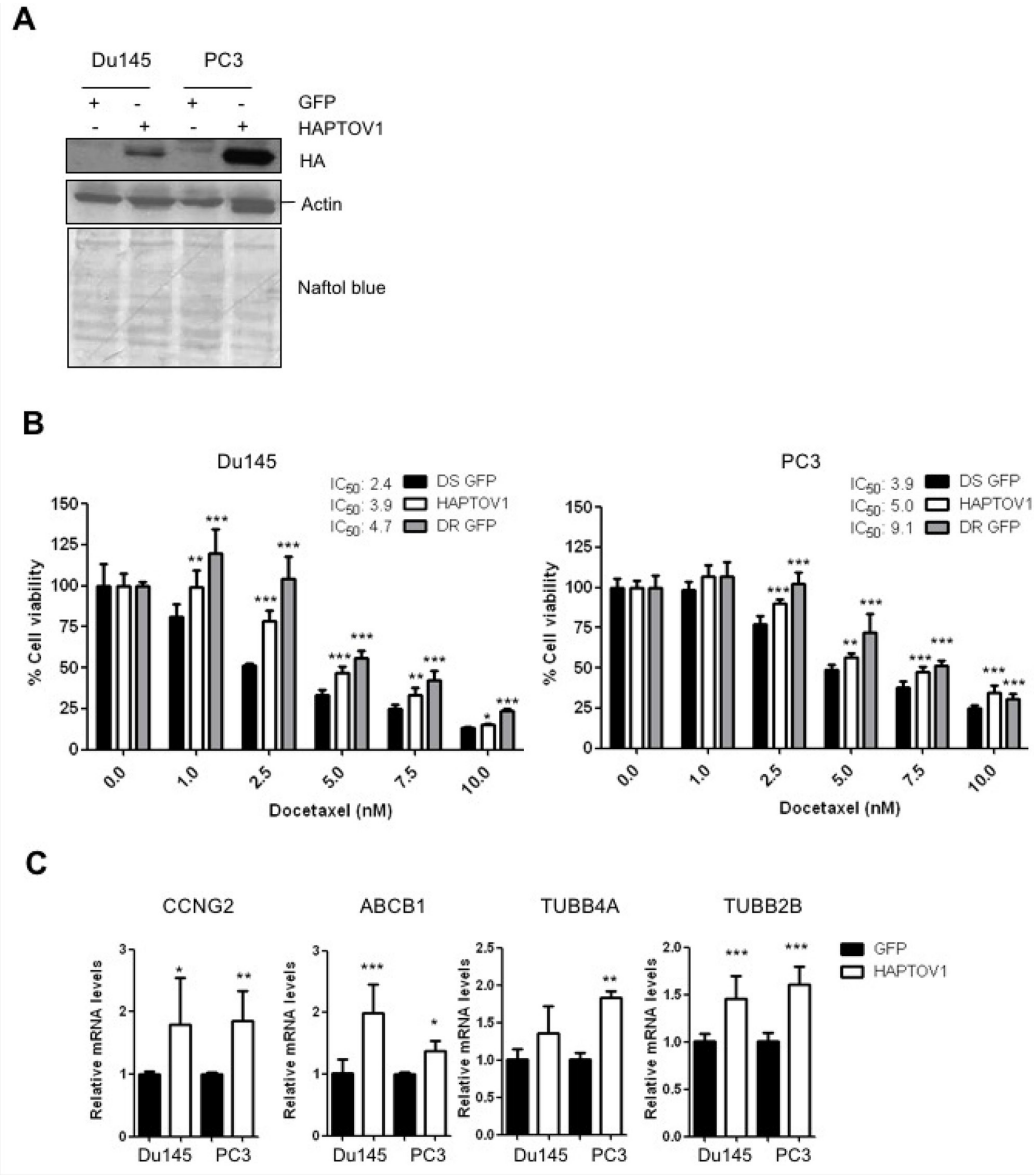
The ectopic expression of PTOV1 in DS Du145 and PC3 cell lines promotes docetaxel resistance **(A)** DS-Du145 or DS-PC3 cells transduced with a lentivirus encoding the fusion protein HA-PTOV1 or a control lentivirus encoding the GFP protein, were analyzed by western blotting using first antibodies to Hemoagglutinin (HA) to detect PTOV1, then antibodies to actin. The double bands in the HAPTOV1 lanes visible in the second panel (actin), are the signal left from the anti-HA antibody. **(B)** Transduced cells were treated with increasing doses of docetaxel for 48 h (Du145) or 72 h (PC3) and cell viability was analyzed by crystal violet. Control docetaxel resistant GFP cells (DR GFP) are shown for reference. A representative experiment of three performed is shown. **(C)** Real-time RT-PCR of the genes indicated analyzed in DS-HAPTOV1 cells compared to controls (DS-GFP). Results are the mean of three independent experiments performed in triplicates and are shown as the mean ± S.D; p-value: *< 0.05; **< 0.01; *** < 0.005. nM, nanomolar.

To establish if the increased PTOV1 expression in DR cells has a role in the acquisition of resistance to docetaxel, DS cells were transduced with a lentivirus encoding HAPTOV1, or a control lentivirus encoding the GFP gene (Figure 2A; Supplementary Figure 3A). Both the endogenous and the ectopic PTOV1 show similar distributions in the membrane, cytoplasm and nucleus (Supplementary Figure 3B). Transduced cells were treated with increasing doses of docetaxel for 48 h (Du145) and 72 h (PC3). The expression of PTOV1 was associated to a significantly augmented IC_50_ to docetaxel in both cell lines, compared to control DS-GFP cells (Figure 2B). The IC_50_ for docetaxel in resistant Du145 and PC3 cells transduced with control lentivirus are also shown for comparison. To elucidate the molecular mechanisms implicated in this PTOV1-mediated chemo resistance, a battery of genes previously implicated in docetaxel resistance were analyzed in PTOV1-overexpressing cells [22, 23, 31, 34]. Figure 2C shows that PTOV1 significantly induces the expression of *CCNG2, ABCB1, TUBB4A* and *TUBB2B* genes, supporting its action in promoting the resistance to docetaxel. The expression of PTOV1 significantly associated with the levels of the multidrug transporter *ABCB1* (Supplementary Figure 3C).

Because upregulation of *ABCB1* and *TUBB2B* were also observed when PTOV1 was overexpressed in the AR-positive LNCaP cells (Supplementary Figure 3D), the PTOV1-induced upregulation of these genes may be AR independent.

In contrast, ectopic PTOV1 in DS-Du145 and DS-PC3 cells exposed to cabazitaxel, a second generation of taxanes synthesized to overcome the resistance to docetaxel, did not produce significant changes in the cells sensitivity to this drug, indicating the specificity of PTOV1 in promoting resistance to docetaxel in CRPC cells (Supplementary Figure 4) [37]. Results also suggest that the induction of *ABCB1* expression might be a relevant part of the mechanism by which PTOV1 confers cells with resistance to docetaxel.

### PTOV1 promotes the acquisition of self-renewing properties in CRPC cells

**Figure 3:**
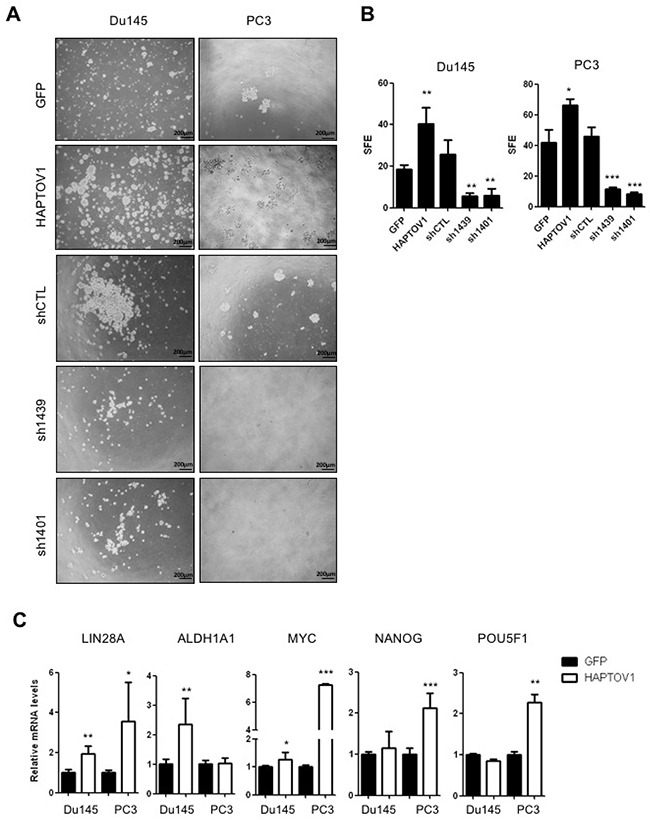
PTOV1 increases the tumorigenic capacity of Du145 and PC3 cells *in vitro* and induces the expression of self-renewal genes **(A)** Phase contrast images, and **(B)** quantification of prostatospheres from cells transduced with lentiviruses encoding HAPTOV1 or GFP, with lentiviruses carrying different shRNA sequences (sh1439, sh1401) to knockdown PTOV1, or a control knockdown lentivirus carrying non-specific sequences (shCTL). Images in A were acquired with an inverted microscope (BX61, Olympus). SFE: Sphere Forming Efficiency (number of spheres formed /1000 cells). **(C)** Relative mRNA levels by real-time RT-PCR of the genes associated to self-renewal in DS-HAPTOV1 and DS-GFP cells. All experiments were performed in triplicate. Results are shown as the mean ± S.D; p-value: *< 0.05; **< 0.01; *** < 0.005.

The significant association between *PTOV1* and *ABCB1* transporter expression, a protein also implicated in the modulation of the stemness phenotype in cancer cells [38], is in agreement with previous data showing PTOV1 promoting self-renewing properties of prostate and breast cancer cells [11, 16]. The use of sphere-forming assays provides a widely recognized *in vitro* method for the identification and characterization of CSCs [39]. Thus, we investigated whether the role of PTOV1 in the resistance to docetaxel is associated to the activation of self-renewing characteristics in prostate cancer cells by analyzing the effects of the overexpression and knockdown of PTOV1 in the prostatosphere forming capacities of docetaxel-sensitive cells. Figure 3A and 3B show that DS-HAPTOV1 cells have a significant increase in the sphere-forming efficiency in comparison to DS-GFP control cells, confirming the PTOV1 promoted induction of a stemness-*like* phenotype. In Du145 cells, the overexpression of HAPTOV1 also associated to significantly increased levels of stemness genes *LIN28A, ALDH1A1* and *MYC*, whereas in PC3 cells it associated to a significant expression of *LIN28A, MYC, NANOG* and *POU5F1* genes (Figure 3C), in agreement with reports in breast cancer cells [16]. Significantly increased levels of *LIN28A* and *MYC* were also observed in LNCaP cells overexpressing HAPTOV1 (Supplementary Figure 5A). Of note, the expression of *PTOV1* is significantly associated with the levels of *LIN28A* in Du145 cells (Supplementary Figure 5B), indicating again its ability to induce a stemness phenotype and confirming previous observations [11, 16].

PTOV1 actions were previously described to be mediated in part through the activation of c-Jun protein synthesis [10] and Wnt/β-catenin signaling [16]. In Du145 cells the inhibition of JNK signaling significantly decreases *ABCB1* expression, as previously described [40] (Supplementary Figure 6A). Regarding Wnt/ β-catenin signaling, these cells require Wnt3a conditioned medium (Wnt3a-CM) for its activation (Supplementary Figure 6B). Wnt3a-CM upregulates *ABCB1*, *ALDH1A1* and Wnt-target genes *LEF1* and *JUN*, effects counteracted by the iCRT14 Wnt inhibitor, as previously observed [41–44] (Supplementary Figure 6B). The overexpression of PTOV1 significantly increased *ABCB1* expression and, reversibly, this was significantly abolished by Wnt and JNK inhibitors, suggesting that PTOV1 action on this gene is mediated at least in part by these pathways (Supplementary Figure 6C). However, no inhibition of *ALDH1A1* or *LIN28A* expression is observed in the presence of these inhibitors in PTOV1-overexpressing cells, suggesting a Wnt and JNK independent actions of PTOV1 (Supplementary Figure 6C).

### The knockdown of PTOV1 induces G_2_/M cell cycle arrest and apoptosis of CRPC cells

**Figure 4:**
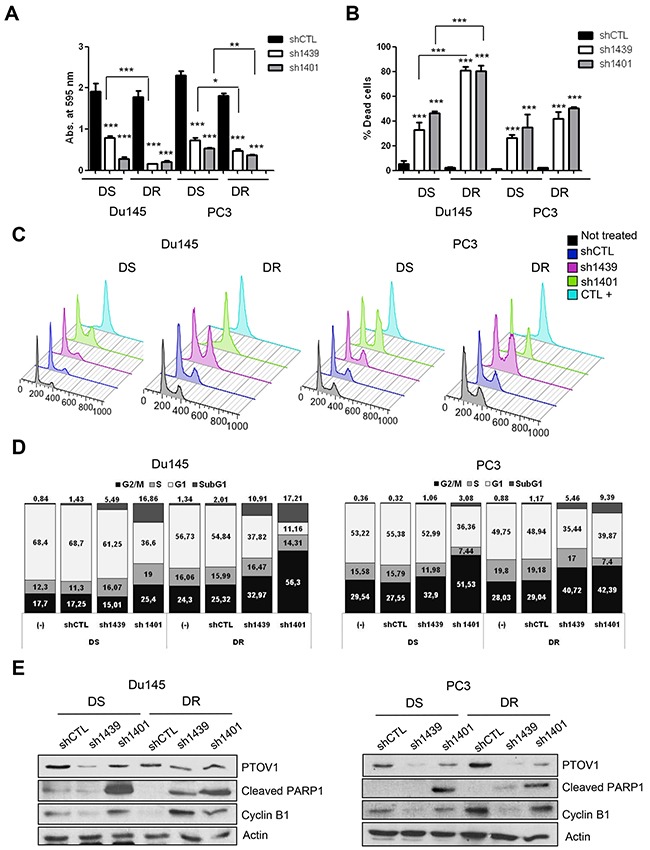
Downregulation of PTOV1 reduces proliferation and promotes G_2_/M cell cycle arrest and apoptosis in CRPC cells Docetaxel sensitive (DS) and resistant (DR) Du145 and PC3 cells were knockdown for PTOV1 using short-hairpin (sh)1439 or sh1401. For coherence in the results and their interpretation, the experiments shown in this figure are from transduced cells at the same passage and same lentiviral preparation. Experiments were reproduced three times with different preparations of lentiviruses with similar results. **(A)** Histogram representing the proliferationof DS and DR Du145 and PC3 cells knockdown for PTOV1 compared to a control shRNA (shCTL). Proliferation was determined by crystal violet staining and absorbance at 595 nm after 96 h from lentiviral transduction. **(B)** Histogram representing the dead cells as assessed by the Trypan blue exclusion in knockdown cells after 96 h from lentiviral transduction. (**C)** Viable knockdown cells and controls as in A, were analyzed at 96 h by flow cytometry using propidium iodide. DS-Du145 cells treated with docetaxel at high concentration (1 μM) for 24 h were used as a control for G2/M arrest (CTL+). **(D)** Histograms representing the percentage of knockdown cells and controls found in each phase of the cell cycle from the experiment shown in C. **(E)** Immunoblots with the indicated antibodies showing the apoptosis caused by the knockdown of PTOV1 compared to controls from the same experiment shown above (A-D). p-value: *< 0.05; **< 0.01; *** < 0.005.

As expected, the knockdown of PTOV1 by different short hairpin RNAs (Supplementary Figure 7A) provokes a striking and significant repressive effect in the cells sphere-forming efficiency (SFE) in comparison to control cells bearing an unrelated shRNA sequence (shCTL) (Figure 3A and 3B). These findings indicate a promoting action of PTOV1 in proliferation and/or survival of prostate cancer cells. Because docetaxel resistant prostate cancer cells show an increased expression of PTOV1 (Figure 1B), we have determined the effects of its knockdown using two different shRNAs directed to different sequences of the gene PTOV1, in both docetaxel sensitive and resistant cells (Supplementary Figure 7A and 7B). RNA and protein expression are efficiently downregulated by these shRNAs, as it is shown by real-time PCR, Western blotting and immunofluorescence assays, the latter showing that downregulation of the protein is observed both in nucleus and cytoplasm (Supplementary Figure 7A-7D). Cell proliferation requires the expression of PTOV1 (Figure 4A). This impair in proliferation is translated in a significant increase in cell mortality, as shown by the Trypan blue exclusion assay (Figure 4B). These effects are enhanced in DR cells compared to DS cells, suggesting that resistant cells are more dependent on the expression of PTOV1. The use of a third shRNA (sh1397) corroborate that PTOV1 inhibition significantly affects cell proliferation and viability (Supplementary Figure 8). The decrease in PTOV1 levels induces a loss of reproductive integrity and inability to proliferate indefinitely, as shown by clonogenic assays (Supplementary Figure 9A and 9B). Of note, docetaxel resistant cells are more enriched with low-proliferative cells compared to sensitive cells, as it is evident from the more abundant formation of smaller colonies.

To investigate into the mechanisms of the requirement for PTOV1 in proliferation and/or survival, the cell cycle was analyzed by flow cytometry using propidium iodide. Figure 4C and 4D show that cells knockdowns for PTOV1 significantly accumulate in the G2/M phase in concomitance to a significant increase in the proportion of cells in the sub-G1 peak (Supplementary Figure 10A and 10B). In agreement with our previous observations, the accumulation of cells in the G2/M phase and the increase in the sub-G1 peak are more pronounced in DR cells (Figure 4C and 4D). The expression of cyclin B1 and the appearance of a cleaved PARP1 fragment in the knockdown cells, confirm the accumulation of cells in G2/M phase and the boost of apoptosis observed by cell cycle analyses (Figure 4E). These results support a requirement of the onco-protein PTOV1 for the survival of Du145 and PC3 prostate cancer cells.

### Correlation of PTOV1 with ALDH1A1 and CCNG2 expression in metastatic prostate tumors

**Figure 5:**
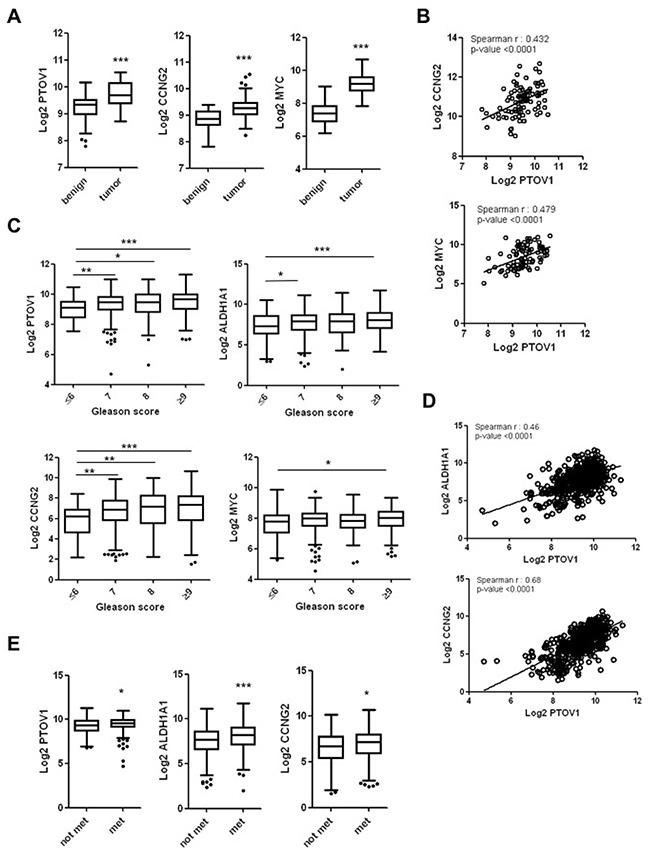
The expression of *PTOV1, ALDH1A1, CCNG2* and *MYC* is significantly increased in human prostate tumors **(A)** Box and whisker plots represent *PTOV1, CCNG2* and *MYC* expression levels in prostate cancer compared to benign tissues from publicly available database (GSE29079; benign n=48; tumor n=47). **(B)** Significant statistical correlations between the expression of *PTOV1* with *CCNG2* and *MYC* in tumors analyzed in A. **(C)** Box and whisker plots representing *PTOV1, ALDH1A1, CCNG2* and *MYC* expression in tumors with different Gleason as obtained from published prostate cancer gene expression profiles (GSE46691; Gleason score (GS) ≤6, n= 63; GS 7, n=271; GS 8, n=68; GS ≥9, n=143). **(D)** Significant statistical correlations between the expression of *PTOV1* with *ALDH1A1* and *CCNG2* in tumors analyzed in C. **(E)** Box and whisker plots representing *PTOV1, ALDH1A1* and *CCNG2* expression in tumors of patients that did not develop metastasis after radical prostatectomy (not met, n=333) compared to patients that did developed metastasis (met, n=212) (GSE46691). p-value: *< 0.05; **< 0.01; *** < 0.005.

The expression of *ALDH1A1, CCNG2* and *MYC* genes has been reported in aggressive prostate tumors [34, 45–48]. We examined the clinical significance of their upregulation and the association with PTOV1, highly overexpressed in aggressive prostate tumors (Supplementary Figure 11) as previously reported [10, 11], using publicly available database containing expression data, clinical and pathological information from prostate tumors of not-treated patients. These analyses reveal that primary prostate tumors show significantly higher expression of *PTOV1, CCNG2* and *MYC* compared to benign tissue (GSE29079), and *PTOV1* levels significantly correlate with *CCNG2* and *MYC* mRNA levels (Figure 5A and 5B). As expected, patients with higher Gleason score have significantly higher levels of *PTOV1* compared to patients with lower Gleason score (GSE46691) (Figure 5C) [9–11]. In this set of tumors, the expression of *ALDH1A1* and *CCNG2* significantly correlates with prostate tumor aggressiveness (Figure 5C), in agreement with previous reports [45, 46]. In addition, a statistically significant correlation exists between the expression of *PTOV1* with *ALDH1A1* and *CCNG2* (Figure 5D). However, the expression of *MYC* appears more significantly associated to the presence of carcinoma compared to benign tissue, than to the aggressiveness of the tumors according to this dataset. *ALDH1A1*, *PTOV1* and *CCNG2* transcripts levels are also significantly higher in primary prostatic adenocarcinomas of patients that after radical prostatectomy developed regional or distal metastasis (Figure 5E), suggesting their relationship with metastatic progression.

The correlation of the overexpression of PTOV1 with tumor progression and poor survival is confirmed in breast cancer [15, 16, 49]. We analyzed dataset from breast cancer tissues derived from patients treated with taxanes plus anthracyclines (GSE28844) [49] and found a significant increase of *PTOV1* and *CCNG2* mRNA levels in patients with lower Miller and Payne grade, corresponding to bad responders to that chemotherapy (Supplementary Figure 12), reinforcing the potential role of PTOV1 and CCNG2 in conferring resistance to chemotherapy with taxanes.

### Analyses of RNA and DNA events from metastasis of castration resistant tumors

**Figure 6:**
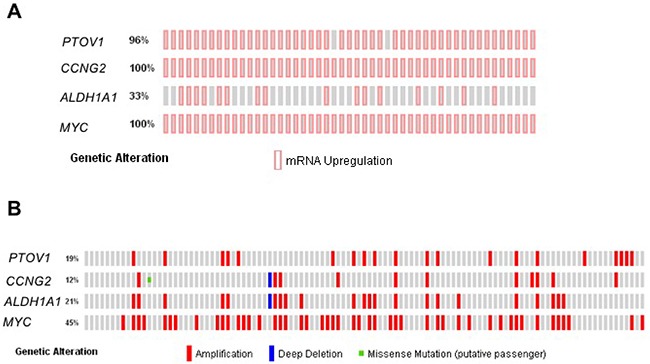
Expression status and mutational profile of PTOV1 and related genes in metastatic lesions from CRPC patients **(A)** Alteration of gene expression in 49 metastatic specimens from 35 CRPC patients (Beltran *et al*. [52]). **(B)** Copy number alteration and mutations in a cohort of 107 metastatic specimens from 77 patients from Beltran *et al*. [52]. Each bar represents one individual. Vertically aligned bars positions correspond to the same specimen.

To study whether the associations of PTOV1 with self-renewal and docetaxel resistant genes found here are detectable in metastasis, we interrogated database containing information from metastatic lesions of prostate cancer patients [50–52]. Forty-nine metastatic lesions from 114 metastatic specimens had available data for RNA expression. Significantly, 96-100% of those lesions show concurrent increased expression of *PTOV1, CCNG2*, and *MYC* genes (Figure 6A). Similarly, concurrent expression is also found for *ALDH1A1* gene in 33% of lesions.

Most alterations at the DNA level cover the complete amplification of PTOV1, amplified in 19% of metastatic specimens (Figure 6B), and include neighboring genes *MED25* and *AKT1S1* (Supplementary Figure 13). Frequent amplifications of *MYC*, *ALDH1A1* and *CCNG2* genes are also found (Figure 6B). In addition, highly significant co-occurrence of alterations in DNA events is found among *PTOV1*, *ALDH1A1*, and *MYC* (Table 1).

**Table 1:** Co-occurrence of DNA alterations in metastatic CRPC

Gene A	Gene B	p-Value	Log odds ratio
*PTOV1*	*ALDH1A1*	**<0.001**	2.338
*CCNG2*	*ALDH1A1*	**<0.001**	2.554
*ALDH1A1*	*MYC*	**<0.001**	>3
*PTOV1*	*MYC*	**0.003**	1.591
*CCNG2*	*MYC*	0.056	1.155
*PTOV1*	*CCNG2*	0.065	1.191

The table shows the quantification (log odds ratio) of how strongly the presence of an alteration in gene A is associated with an alteration in the gene B analyzed from published datasets from Beltran *et al*. [52]. Log odds>0: association towards co-occurrence. *p*-value <0.05.

Altogether, our findings indicate that the overexpression of PTOV1 in prostate cancer promotes the acquisition of resistance to chemotherapy with docetaxel that is parallel to the increase in self-renewal properties of prostate cancer cells. The significant increased expression of *PTOV1* in primary prostate tumors may be predictor of metastastatic progression.

## DISCUSSION

The oncogenic protein PTOV1 is associated to bad prognosis in several tumor types [8–11, 14, 15, 17, 18, 53–55]. In this work we provide evidences supporting its role in promoting the resistance to docetaxel and self-renewal abilities of prostate cancer cells. We further show that the expression of *PTOV1, ALDH1A1* and *CCNG2* found in primary prostate tumors of patients that had developed metastasis, is also found in a cohort of metastasic lesions, revealing that the above genes may be predictors of metastasis and bad prognosis.

Supporting previous findings of PTOV1 activity associated to more aggressive, potentially resistant stages [8–11], we show that docetaxel resistant prostate cancer cells express increased levels of PTOV1 concomitantly with genes associated to resistance [31, 33–36]. The PTOV1 induced expression in docetaxel sensitive cells, significantly increased their IC_50_ to this drug and activated the expression of *ABCB1*, *TUBB4A*, *TUBB2B, MYC*, and *CCNG2* genes, demonstrating PTOV1 as a relevant factor to overcome toxicity to docetaxel. The PTOV1-promoted increase of *ABCB1* expression, a drug transporter with high affinity for docetaxel but low affinity for cabazitaxel [37, 56], might explain its specific action in inducing cell resistance to docetaxel but not to cabazitaxel. The upregulation of *ABCB1* might be a major mechanism of taxane resistance in the early response to docetaxel in advanced tumors, and in Du145, 22Rv1 and C4-2B prostate cancer cell lines [22, 23, 36, 57]. However, the expression of the protein ABCB1, not its RNA levels, was shown to correlate with the Gleason score in prostate tumors, possibly explained by a lower efficiency of RNA evaluation in those samples [58]. Recently, PTOV1 was shown to increase the proportion of cells in the side population (SP) through the activation of Wnt/β-catenin signaling in breast cancer [16]. Several studies described SP cells as CSC populations with upregulation of ABCB1 and a taxol-resistant phenotype [59–61]. These observations support our findings and suggest that PTOV1 promotes the acquisition of resistance to docetaxel and of stemness features partly through the expression of *ABCB1*. Although the specific increase of stemenss genes *ALDH1A1* and *LIN28A* by PTOV1 does not appear mediated by Wnt and JNK signaling, the PTOV1 promoted *ABCB1* activation might be mediated by these pathways.

The concurrent overexpression of *CCNG2* and *ALDH1A1* found in the cell lines with the ectopic HAPTOV1 is confirmed in primary tumors of patients that developed metastasis and in metastatic lesions of CRPC patients. While *ALDH1A1* and *MYC* genes were reported in metastasis of several tumor types and associated with poor prognosis [39, 45, 47, 62, 63], for CCNG2, a cell cycle checkpoint cyclin upregulated in response to DNA damage, the association to stemness was not clear. It was reported to have tumor suppressor functions in different neoplasias [64, 65], although, supporting our observations, CCNG2 positively associated with prostate cancer recurrence after radical prostatectomy [46]. CCNG2 induces microtubule bundling and stability, and contributes to resistance to chemotherapy, presumably by facilitating a delay in the transition from G1/S to G2/M phase that allow reorganization of microtubules and chromatin repairs after drug exposure [34, 66–68].

The development of CRPC was described to lead to increased resistance to docetaxel also in chemotherapy-naïve patients, suggesting that an intrinsic chemoresistance may be present in tumors even before drug exposure [69–71]. Chemotherapy effectively induces death of the bulk of proliferating tumor cells and concomitantly results in the enrichment of cell populations characterized by a higher intrinsic resistance, such as CSCs, considered major responsible for the poor prognosis of patients with cancer [25, 69, 72]. In line with this possibility, our findings suggest that aggressive tumor cells with increased expression of *PTOV1*, *ALDH1A1*, and *CCNG2* might correspond to potential CSCs with great capacity to metastasize and higher resistance to docetaxel.

The analyses of public datasets containing data from untreated patients, do not confirm the statistically significant correlation of *PTOV1* with *ABCB1, TUBB4A, TUBB2B, NANOG, POU5F1*, and *LIN28A* found in the cell line models. This lack of correlation might be related to the metastatic origin of the cell lines from CRPC patients, a more advanced tumor stage compared to the untreated tumors included in the data base [31, 73]. In addition, the known heterogeneous genetic profiles of prostate tumors may not allow to discern those alterations found in isolated cells, that may be present in specific subsets of tumors but become undetectable in a large heterogeneous cohort [58, 74].

PTOV1 action was previously found associated to cell cycle progression of prostate cancer cells [9]. Here, we show its critical role for the survival of docetaxel-resistant cells. PTOV1 is an activator of protein synthesis and its function might be crucial in the G2 phase of the cell cycle before mitosis when there is a need for a rapid synthesis of proteins required for cell division [10]. In addition, the activation of Snail1 expression by PTOV1 [10] might be linked to its action in the development of resistance to docetaxel [29, 30]. These results support that cells arrested at the end of the G2 phase in PTOV1 knockdowns are unable to bypass the DNA damage checkpoint and enter a programmed cell death.

All together our findings indicate that *PTOV1* confers prostate cancer cells the advantages to survive docetaxel toxicity, through upregulation of *ABCB1* and genes associated to docetaxel resistance and pluripotency factors. Our study identifies *PTOV1, ALDH1A1* and *CCNG2* as potential markers of metastasis and bad prognosis when detected in primary prostate tumors. Although further validations are required in additional models, our data reveal that blocking PTOV1 might be a valid future option to prevent the development of resistance to docetaxel in CRPC.

## MATERIALS AND METHODS

### Cell lines, cell cultures and reagents

HEK293T, human prostate adenocarcinoma LNCaP, Du145 and PC3 cells lines were purchased from the American Type Culture Collection, Manassas, VA, USA. The docetaxel resistant cell lines Du145 and PC3 were developed as previously described [31]. HEK293T were cultured in DMEM (BioWest, Nuaillé, France), docetaxel sentitive and resistant Du145 cells and LNCaP cells were cultured in RPMI-1640 (BioWest) and PC3 DS and PC3 DR in F12K nutrient mixture medium (Thermo Fisher Scientific, Waltham, MA, USA). Culture media were supplemented with 10% fetal bovine serum (FBS), 2 mM l-glutamine, 100 U of penicillin/ml, 100 μg of streptomycin/ml, and 0.1 mM non-essential amino acids (all from BioWest). Docetaxel resistant cell lines were maintained with 2.5 nM of Docetaxel (Sigma-Aldrich, St. Louis, MO). Antibody to PTOV1 was produced and purified as previously described [8, 9]. Additional antibodies were obtained from: goat anti-actin (I-19) (Santa Cruz Biotechnology, Santa Cruz, CA); mouse anti-HA.11 (16B12) (Covance, MA); mouse anti-Cyclin B1(V152) (Abcam, Cambridge, MA); rabbit anti-PARP1 (H-250) (Santa Cruz Biotechnology). Lentiviral vectors carrying short-hairpin RNA (shRNA, TRCN0000143905, TRCN0000140104 and TRCN0000139737) to PTOV1 were obtained from Sigma.

### Transfections and transduction

Lentiviral particles were produced in HEK293T by cotransfection of lentiviral vectors carrying short hairpin RNA (shRNA) or HAPTOV1 templates and plasmids encoding gag, pol and env proteins as previously described [10].

### Plasmids

The lentiviral pHAPTOV1-ires-GFP vector was generated by replacing the *Luciferase* from with Hemagglutinin (HA)-tagged human PTOV1, as previously described [10]

### Protein stability analysis

Cells (3×10^5^ cells/well) were seeded on 6 well plates. After 24 h, cells were treated with complete medium supplemented with 100 μg/ml cycloheximide (Sigma-Aldrich) at indicated times (1, 3, 6, 9 or 15 h). Protein extraction and western blotting analysis were performed as previously described [53].

### Citotoxicity assays

Cells (4×10^3^ cells/well) were seeded on collagen-coated 96-well plates in sextuplicates. After 24 h, cells were control-treated (DMSO) or treated with the drug at different concentrations for 48 h for Du145 cells and 72 h for PC3 cells. Fresh medium with the drug was replaced every day. At the indicated time points, cells were fixed in formaldehyde 4% solution, washed with PBS and stained with 0.5% crystal violet. Crystals were dissolved with 15% acetic acid and optical density was read at 595 nm.

### Viability assays

Cells, transducted with lentiviral particles during 24 h, were seeded (5×10^4^ cells/well) on 24-well plates in triplicates. After 72 h, cells were collected and stained with Trypan blue to identify dead cells. Alive and dead cells were counted in a Neubauer chamber.

### Spheres formation assay

Single-cells derived from monolayer cultures were seeded (3 × 10^3^ cells/well) in 24-well Ultra Low Attachment culture plates (Corning Costar, Cambridge, MA) in triplicates in 1 ml of DMEM-F12 culture medium (Invitrogen) containing 1% methyl cellulose, 60 μg/ml glucose, 1 μM hydrocortisone, 1 μg/ml putrescine, 10 μg/ml transferrin, 3 nM sodium selenite, 2.5 ug/ml insulin, 10 ng/ml β-FGF, 20 ng/ul, EGF5 U.I./ml heparin (Sigma-Aldrich) and 0.4% B27 (Invitrogen). Prostatospheres were fed every two days and allowed to grow for 14 days. The number of spheres (> 50 cells/sphere) were then counted and expressed as ratio of spheres/1000 plated cells (Sphere Forming Efficiency, SFE).

### Real-time PCR

Total RNA was extracted with the RNeasy mini kit (QIAGEN, Valencia, CA), reverse transcribed with the RevertAid H Minus First Strand cDNA Synthesis kit (ThermoScientific) and real-time qPCR performed with the Universal Probe Library (Roche, Penzberg, Upper Bavaria, Germany) on a LightCycler 480 RealTime PCR instrument (Roche). Primers are shown in Supplementary Table 1. The ΔΔCt method was applied to estimate relative transcript levels. HMBS amplification levels were used as internal references. β-actin amplification levels were used as internal references for real-time PCR of polysomes-bounded RNA. At least three independent experiments with triplicate samples were performed. Values are presented as mean + SD.

### DNA and mRNA analyses in human samples

The mRNA expression data of several genes was analyzed in dataset from human prostate tumors (GSE46691, n=545; GSE2907, n=95) and breast tumors (GSE28844, n=61) using the R2 bioinformatics platform (http://r2.amc.nl). The most informative probeset, according to its average present signal (APS) and average value (Avg) was selected in every dataset, sometimes resulting in a different probeset as the choice of R2. DNA amplification, point mutations, deletions of the genes of interest were analyzed from published datasets of human prostate tumors using cBioPortal platform [50, 51].

### Western blotting

Western blotting analyses were performed as previously described [53]. Specific reactivity to antibodies was detected with a chemiluminescent substrate (GE Healthcare, Solingen, Germany). Actin or tubulin signal was used to monitor protein loading and transfer efficiencies.

### Cell cycle analysis

One million cells were collected by trypsinization and washed twice with PBS. After centrifugation 5 min at 300 xG, pellets were resuspended and fixed with 70% cold ethanol. Cells were stored overnight at 4°C. Next, cells were pelleted by centrifugation, washed twice with cold PBS and resuspended in staining solution, 1 mM sodium citrate, 15 μg/ml propidium iodide and 300 μg/ml DNAse free RNase A and incubated 4°C O/N. The samples (1 ×10^6^ cells) were analyzed by flow cytometry with a BD FacsCalibur device and FlowJo software.

### Statistics

All data analyzed were from at least triplicate experiments. Results are expressed as means + standard deviation of the means. IC_50_ values for docetaxel treatment were determined by non-linear regression. For statistical analysis, according to whether data were sampled from a Gaussian distribution or not, unpaired t test or Mann-Whitney U test was used to compare two groups. For multiple comparisons, ANOVA followed by Dunnett method or the alternative non parametric method (Kruskal-Wallis followed by Dunn method) were employed. To quantify the association between two quantitative variables, the Spearman correlation was used for statistical analysis. The Fisher's exact test was employed to determine whether a nonrandom association of co-occurrence between two categorical variables was significant. A *p* value < 0.05 was taken as the level of significance. These analyses were performed using GraphPad Prism 5 software.

## SUPPLEMENTARY MATERIALS FIGURES AND TABLE



## Abbreviations

ADT: androgen deprivation therapy
ALDH1A1: aldehyde dehydrogenase 1 family member A1
AR: androgen receptor
CCNG2: cyclin G2
CRPC: castration resistant prostate cancer
CSC: cancer stem cell
DKK1: dickkopf WNT signaling pathway inhibitor 1
DR: docetaxel resistant
DS: docetaxel sensitive
EMT: epithelial-mesenchymal transition
HES1: hes family bHLH transcription factor 1
HEY1: hes related family bHLH transcription factor with YRPW motif 1
JNK: c-Jun N-terminal Kinase
PARP1: poly ADP-ribose polymerase-1
PTOV1: prostate tumor overexpressed 1
RACK1: receptor for activated C kinase 1
RARβ: retinoic acid receptor β
SFE: sphere forming efficiency.

## References

[1] Siegel RL, Miller KD, Jemal A (2016). Cancer statistics, 2016. CA Cancer J Clin.

[2] Cornford P, Bellmunt J, Bolla M, Briers E, De Santis M, Gross T, Henry AM, Joniau S, Lam TB, Mason MD, van der Poel HG, van der Kwast TH, Rouviere O (2017). EAU-ESTRO-SIOG guidelines on prostate cancer. Part II: Treatment of relapsing, metastatic, and castration-resistant prostate cancer. Eur Urol.

[3] Chandrasekar T, Yang JC, Gao AC, Evans CP (2015). Mechanisms of resistance in castration-resistant prostate cancer (CRPC). Transl Androl Urol.

[4] Petrylak DP, Tangen CM, Hussain MH, Lara PN, Jones JA, Taplin ME, Burch PA, Berry D, Moinpour C, Kohli M, Benson MC, Small EJ, Raghavan D, Crawford ED (2004). Docetaxel and estramustine compared with mitoxantrone and prednisone for advanced refractory prostate cancer. N Engl J Med.

[5] Tassinari D, Tamburini E, Gianni L, Drudi F, Fantini M, Santelmo C, Stocchi L, Montanari F, Sartori S (2016). Early docetaxel and androgen deprivation in the treatment of metastatic, hormone-sensitive prostate cancer. Rev Recent Clin Trials.

[6] James ND, Sydes MR, Clarke NW, Mason MD, Dearnaley DP, Spears MR, Ritchie AW, Parker CC, Russell JM, Attard G, de Bono J, Cross W, Jones RJ (2016). Addition of docetaxel, zoledronic acid, or both to first-line long-term hormone therapy in prostate cancer (STAMPEDE): survival results from an adaptive, multiarm, multistage, platform randomised controlled trial. Lancet.

[7] Gravis G, Boher JM, Joly F, Soulie M, Albiges L, Priou F, Latorzeff I, Delva R, Krakowski I, Laguerre B, Rolland F, Theodore C, Deplanque G (2016). Androgen deprivation therapy (ADT) plus docetaxel versus ADT alone in metastatic non castrate prostate cancer: impact of metastatic burden and long-term survival analysis of the randomized phase 3 GETUG-AFU15 trial. Eur Urol.

[8] Benedit P, Paciucci R, Thomson TM, Valeri M, Nadal M, Caceres C, de Torres I, Estivill X, Lozano JJ, Morote J, Reventos J (2001). PTOV1, a novel protein overexpressed in prostate cancer containing a new class of protein homology blocks. Oncogene.

[9] Santamaria A, Fernandez PL, Farre X, Benedit P, Reventos J, Morote J, Paciucci R, Thomson TM (2003). PTOV-1, a novel protein overexpressed in prostate cancer, shuttles between the cytoplasm and the nucleus and promotes entry into the S phase of the cell division cycle. Am J Pathol.

[10] Marques N, Sese M, Canovas V, Valente F, Bermudo R, de Torres I, Fernandez Y, Abasolo I, Fernandez PL, Contreras H, Castellon E, Celia-Terrassa T, Mendez R (2014). Regulation of protein translation and c-Jun expression by prostate tumor overexpressed 1. Oncogene.

[11] Alana L, Sese M, Canovas V, Punyal Y, Fernandez Y, Abasolo I, de Torres I, Ruiz C, Espinosa L, Bigas A, Y Cajal SR, Fernandez PL, Serras F (2014). Prostate tumor OVerexpressed-1 (PTOV1) down-regulates HES1 and HEY1 notch targets genes and promotes prostate cancer progression. Mol Cancer.

[12] Mazzucchelli R, Scarpelli M, Barbisan F, Santinelli A, Lopez-Beltran A, Cheng L, Montironi R (2013). Immunohistochemical expression of prostate tumour overexpressed 1 (PTOV1) in atypical adenomatous hyperplasia (AAH) of the prostate: additional evidence linking (AAH) to adenocarcinoma. Cell Oncol (Dordr).

[13] Morote J, Fernandez S, Alana L, Iglesias C, Planas J, Reventos J, Ramon YC, Paciucci R, de Torres IM (2008). PTOV1 expression predicts prostate cancer in men with isolated high-grade prostatic intraepithelial neoplasia in needle biopsy. Clin Cancer Res.

[14] Chen SP, Zhang LS, Fu BS, Zeng XC, Yi HM, Jiang N (2015). Prostate tumor overexpressed 1 is a novel prognostic marker for hepatocellular carcinoma progression and overall patient survival. Medicine.

[15] Lei F, Zhang L, Li X, Lin X, Wu S, Li F, Liu J (2014). Overexpression of prostate tumor overexpressed 1 correlates with tumor progression and predicts poor prognosis in breast cancer. BMC Cancer.

[16] Cui Y, Ma W, Lei F, Li Q, Su Y, Lin X, Lin C, Zhang X, Ye L, Wu S, Li J, Yuan Z, Song L (2016). Prostate tumour overexpressed-1 promotes tumourigenicity in human breast cancer via activation of Wnt/beta-catenin signalling. J Pathol.

[17] Fernandez S, Mosquera JL, Alana L, Sanchez-Pla A, Morote J, Ramon YC, Reventos J, de Torres I, Paciucci R (2011). PTOV1 is overexpressed in human high-grade malignant tumors. Virchows Arch.

[18] Yang L, Wang H, Wang Y, He Z, Chen H, Liang S, He S, Wu S, Song L, Chen Y (2016). Prostate tumor overexpressed-1 in conjunction with human papillomavirus status, predicts outcome in early-stage human laryngeal squamous cell carcinoma. Oncotarget.

[19] Youn HS, Park UH, Kim EJ, Um SJ (2011). PTOV1 antagonizes MED25 in RAR transcriptional activation. Biochem Biophys Res Commun.

[20] Canovas V, Lleonart M, Morote J, Paciucci R (2017). The role of prostate tumor overexpressed 1 in cancer progression. Oncotarget.

[21] Shalli K, Brown I, Heys SD, Schofield AC (2005). Alterations of beta-tubulin isotypes in breast cancer cells resistant to docetaxel. FASEB J.

[22] Hansen SN, Westergaard D, Thomsen MB, Vistesen M, Do KN, Fogh L, Belling KC, Wang J, Yang H, Gupta R, Ditzel HJ, Moreira J, Brunner N (2015). Acquisition of docetaxel resistance in breast cancer cells reveals upregulation of ABCB1 expression as a key mediator of resistance accompanied by discrete upregulation of other specific genes and pathways. Tumour Biol.

[23] Domingo-Domenech J, Vidal SJ, Rodriguez-Bravo V, Castillo-Martin M, Quinn SA, Rodriguez-Barrueco R, Bonal DM, Charytonowicz E, Gladoun N, de la Iglesia-Vicente J, Petrylak DP, Benson MC, Silva JM, Cordon-Cardo C (2012). Suppression of acquired docetaxel resistance in prostate cancer through depletion of notch- and hedgehog-dependent tumor-initiating cells. Cancer Cell.

[24] Oskarsson T, Batlle E, Massague J (2014). Metastatic stem cells: sources, niches, and vital pathways. Cell Stem Cell.

[25] Lu CS, Shieh GS, Wang CT, Su BH, Su YC, Chen YC, Su WC, Wu P, Yang WH, Shiau AL, Wu CL (2017). Chemotherapeutics-induced Oct4 expression contributes to drug resistance and tumor recurrence in bladder cancer. Oncotarget.

[26] Archer LK, Frame FM, Maitland NJ (2017). Stem cells and the role of ETS transcription factors in the differentiation hierarchy of normal and malignant prostate epithelium. J Steroid Biochem Mol Biol.

[27] Thadani-Mulero M, Portella L, Sun S, Sung M, Matov A, Vessella RL, Corey E, Nanus DM, Plymate SR, Giannakakou P (2014). Androgen receptor splice variants determine taxane sensitivity in prostate cancer. Cancer Res.

[28] Darshan MS, Loftus MS, Thadani-Mulero M, Levy BP, Escuin D, Zhou XK, Gjyrezi A, Chanel-Vos C, Shen R, Tagawa ST, Bander NH, Nanus DM, Giannakakou P (2011). Taxane-induced blockade to nuclear accumulation of the androgen receptor predicts clinical responses in metastatic prostate cancer. Cancer Res.

[29] Kurrey NK, Jalgaonkar SP, Joglekar AV, Ghanate AD, Chaskar PD, Doiphode RY, Bapat SA (2009). Snail and slug mediate radioresistance and chemoresistance by antagonizing p53-mediated apoptosis and acquiring a stem-like phenotype in ovarian cancer cells. Stem Cells.

[30] Tinzl M, Chen B, Chen SY, Semenas J, Abrahamsson PA, Dizeyi N (2013). Interaction between c-jun and androgen receptor determines the outcome of taxane therapy in castration resistant prostate cancer. PLoS One.

[31] Marin-Aguilera M, Codony-Servat J, Kalko SG, Fernandez PL, Bermudo R, Buxo E, Ribal MJ, Gascon P, Mellado B (2012). Identification of docetaxel resistance genes in castration-resistant prostate cancer. Mol Cancer Ther.

[32] Marin-Aguilera M, Codony-Servat J, Reig O, Lozano JJ, Fernandez PL, Pereira MV, Jimenez N, Donovan M, Puig P, Mengual L, Bermudo R, Font A, Gallardo E (2014). Epithelial-to-mesenchymal transition mediates docetaxel resistance and high risk of relapse in prostate cancer. Mol Cancer Ther.

[33] Atjanasuppat K, Lirdprapamongkol K, Jantaree P, Svasti J (2015). Non-adherent culture induces paclitaxel resistance in H460 lung cancer cells via ERK-mediated up-regulation of betaIVa-tubulin. Biochem Biophys Res Commun.

[34] Desarnaud F, Geck P, Parkin C, Carpinito G, Makarovskiy AN (2011). Gene expression profiling of the androgen independent prostate cancer cells demonstrates complex mechanisms mediating resistance to docetaxel. Cancer Biol Ther.

[35] Bernard-Marty C, Treilleux I, Dumontet C, Cardoso F, Fellous A, Gancberg D, Bissery MC, Paesmans M, Larsimont D, Piccart MJ, Di Leo A (2002). Microtubule-associated parameters as predictive markers of docetaxel activity in advanced breast cancer patients: results of a pilot study. Clin Breast Cancer.

[36] Zhu Y, Liu C, Nadiminty N, Lou W, Tummala R, Evans CP, Gao AC (2013). Inhibition of ABCB1 expression overcomes acquired docetaxel resistance in prostate cancer. Mol Cancer Ther.

[37] Abidi A (2013). Cabazitaxel: a novel taxane for metastatic castration-resistant prostate cancer-current implications and future prospects. J Pharmacol Pharmacother.

[38] Huang B, Fu SJ, Fan WZ, Wang ZH, Chen ZB, Guo SJ, Chen JX, Qiu SP (2016). PKCepsilon inhibits isolation and stemness of side population cells via the suppression of ABCB1 transporter and PI3K/Akt, MAPK/ERK signaling in renal cell carcinoma cell line 769P. Cancer Lett.

[39] Le Magnen C, Bubendorf L, Rentsch CA, Mengus C, Gsponer J, Zellweger T, Rieken M, Thalmann GN, Cecchini MG, Germann M, Bachmann A, Wyler S, Heberer M, Spagnoli GC (2013). Characterization and clinical relevance of ALDHbright populations in prostate cancer. Clin Cancer Res.

[40] Sui H, Fan ZZ, Li Q (2012). Signal transduction pathways and transcriptional mechanisms of ABCB1/Pgp-mediated multiple drug resistance in human cancer cells. J Int Med Res.

[41] Correa S, Binato R, Du Rocher B, Castelo-Branco MT, Pizzatti L, Abdelhay E (2012). Wnt/beta-catenin pathway regulates ABCB1 transcription in chronic myeloid leukemia. BMC Cancer.

[42] Condello S, Morgan CA, Nagdas S, Cao L, Turek J, Hurley TD, Matei D (2015). β-Catenin-regulated ALDH1A1 is a target in ovarian cancer spheroids. Oncogene.

[43] Filali M, Cheng N, Abbott D, Leontiev V, Engelhardt JF (2002). Wnt-3A/beta-catenin signaling induces transcription from the LEF-1 promoter. J Biol Chem.

[44] Hwang SG, Yu SS, Lee SW, Chun JS (2005). Wnt-3a regulates chondrocyte differentiation via c-Jun/AP-1 pathway. FEBS Lett.

[45] Kalantari E, Saadi FH, Asgari M, Shariftabrizi A, Roudi R, Madjd Z (2016). Increased expression of ALDH1A1 in prostate cancer is correlated with tumor aggressiveness: a tissue microarray study of Iranian patients. Appl Immunohistochem Mol Morphol.

[46] Long Q, Johnson BA, Osunkoya AO, Lai YH, Zhou W, Abramovitz M, Xia M, Bouzyk MB, Nam RK, Sugar L, Stanimirovic A, Williams DJ, Leyland-Jones BR (2011). Protein-coding and microRNA biomarkers of recurrence of prostate cancer following radical prostatectomy. Am J Pathol.

[47] Hubbard GK, Mutton LN, Khalili M, McMullin RP, Hicks JL, Bianchi-Frias D, Horn LA, Kulac I, Moubarek MS, Nelson PS, Yegnasubramanian S, De Marzo AM, Bieberich CJ (2016). Combined MYC activation and Pten loss are sufficient to create genomic instability and lethal metastatic prostate cancer. Cancer Res.

[48] Ross-Adams H, Lamb AD, Dunning MJ, Halim S, Lindberg J, Massie CM, Egevad LA, Russell R, Ramos-Montoya A, Vowler SL, Sharma NL, Kay J, Whitaker H (2015). Integration of copy number and transcriptomics provides risk stratification in prostate cancer: a discovery and validation cohort study. EBioMedicine.

[49] Vera-Ramirez L, Sanchez-Rovira P, Ramirez-Tortosa CL, Quiles JL, Ramirez-Tortosa M, Lorente JA (2013). Transcriptional shift identifies a set of genes driving breast cancer chemoresistance. PLoS One.

[50] Cerami E, Gao J, Dogrusoz U, Gross BE, Sumer SO, Aksoy BA, Jacobsen A, Byrne CJ, Heuer ML, Larsson E, Antipin Y, Reva B, Goldberg AP (2012). The cBio cancer genomics portal: an open platform for exploring multidimensional cancer genomics data. Cancer Discov.

[51] Gao J, Aksoy BA, Dogrusoz U, Dresdner G, Gross B, Sumer SO, Sun Y, Jacobsen A, Sinha R, Larsson E, Cerami E, Sander C, Schultz N (2013). Integrative analysis of complex cancer genomics and clinical profiles using the cBioPortal. Sci Signal.

[52] Beltran H, Prandi D, Mosquera JM, Benelli M, Puca L, Cyrta J, Marotz C, Giannopoulou E, Chakravarthi BV, Varambally S, Tomlins SA, Nanus DM, Tagawa ST (2016). Divergent clonal evolution of castration-resistant neuroendocrine prostate cancer. Nat Med.

[53] Santamaria A, Castellanos E, Gomez V, Benedit P, Renau-Piqueras J, Morote J, Reventos J, Thomson TM, Paciucci R (2005). PTOV1 enables the nuclear translocation and mitogenic activity of flotillin-1, a major protein of lipid rafts. Mol Cell Biol.

[54] Guo F, Feng L, Hu JL, Wang ML, Luo P, Zhong XM, Deng AM (2015). Increased PTOV1 expression is related to poor prognosis in epithelial ovarian cancer. Tumour Biol.

[55] Reva B, Antipin Y, Sander C (2011). Predicting the functional impact of protein mutations: application to cancer genomics. Nucleic Acids Res.

[56] Duran GE, Wang YC, Francisco EB, Rose JC, Martinez FJ, Coller J, Brassard D, Vrignaud P, Sikic BI (2015). Mechanisms of resistance to cabazitaxel. Mol Cancer Ther.

[57] Karatas OF, Guzel E, Duz MB, Ittmann M, Ozen M (2016). The role of ATP-binding cassette transporter genes in the progression of prostate cancer. Prostate.

[58] Bhangal G, Halford S, Wang J, Roylance R, Shah R, Waxman J (2000). Expression of the multidrug resistance gene in human prostate cancer. Urol Oncol.

[59] Murota Y, Tabu K, Taga T (2016). Requirement of ABC transporter inhibition and Hoechst 33342 dye deprivation for the assessment of side population-defined C6 glioma stem cell metabolism using fluorescent probes. BMC Cancer.

[60] Eyre R, Harvey I, Stemke-Hale K, Lennard TW, Tyson-Capper A, Meeson AP (2014). Reversing paclitaxel resistance in ovarian cancer cells via inhibition of the ABCB1 expressing side population. Tumour Biol.

[61] Wang Y, Teng JS (2016). Increased multi-drug resistance and reduced apoptosis in osteosarcoma side population cells are crucial factors for tumor recurrence. Exp Ther Med.

[62] Li T, Su Y, Mei Y, Leng Q, Leng B, Liu Z, Stass SA, Jiang F (2010). ALDH1A1 is a marker for malignant prostate stem cells and predictor of prostate cancer patients’ outcome. Lab Invest.

[63] Nowak DG, Cho H, Herzka T, Watrud K, DeMarco DV, Wang VM, Senturk S, Fellmann C, Ding D, Beinortas T, Kleinman D, Chen M, Sordella R (2015). MYC drives Pten/Trp53-deficient proliferation and metastasis due to IL6 secretion and AKT suppression via PHLPP2. Cancer Discov.

[64] Hasegawa S, Nagano H, Konno M, Eguchi H, Tomokuni A, Tomimaru Y, Wada H, Hama N, Kawamoto K, Kobayashi S, Marubashi S, Nishida N, Koseki J (2015). Cyclin G2: a novel independent prognostic marker in pancreatic cancer. Oncol Lett.

[65] Sun GG, Zhang J, Hu WN (2014). CCNG2 expression is downregulated in colorectal carcinoma and its clinical significance. Tumour Biol.

[66] Li Y, Hussain M, Sarkar SH, Eliason J, Li R, Sarkar FH (2005). Gene expression profiling revealed novel mechanism of action of Taxotere and Furtulon in prostate cancer cells. BMC Cancer.

[67] Li Y, Hong X, Hussain M, Sarkar SH, Li R, Sarkar FH (2005). Gene expression profiling revealed novel molecular targets of docetaxel and estramustine combination treatment in prostate cancer cells. Mol Cancer Ther.

[68] Arachchige Don AS, Dallapiazza RF, Bennin DA, Brake T, Cowan CE, Horne MC (2006). Cyclin G2 is a centrosome-associated nucleocytoplasmic shuttling protein that influences microtubule stability and induces a p53-dependent cell cycle arrest. Exp Cell Res.

[69] Maroto P, Solsona E, Gallardo E, Mellado B, Morote J, Arranz JA, Gomez-Veiga F, Unda M, Climent MA, Alcaraz A (2016). Expert opinion on first-line therapy in the treatment of castration-resistant prostate cancer. Crit Rev Oncol Hematol.

[70] Rybak AP, He L, Kapoor A, Cutz JC, Tang D (2011). Characterization of sphere-propagating cells with stem-like properties from DU145 prostate cancer cells. Biochim Biophys Acta.

[71] Vaishampayan U, Shevrin D, Stein M, Heilbrun L, Land S, Stark K, Li J, Dickow B, Heath E, Smith D, Fontana J (2015). Phase II trial of carboplatin, everolimus, and prednisone in metastatic castration-resistant prostate cancer pretreated with docetaxel chemotherapy: a prostate cancer clinical trial consortium study. Urology.

[72] Wiechert A, Saygin C, Thiagarajan PS, Rao VS, Hale JS, Gupta N, Hitomi M, Nagaraj AB, DiFeo A, Lathia JD, Reizes O (2016). Cisplatin induces stemness in ovarian cancer. Oncotarget.

[73] Erho N, Crisan A, Vergara IA, Mitra AP, Ghadessi M, Buerki C, Bergstralh EJ, Kollmeyer T, Fink S, Haddad Z, Zimmermann B, Sierocinski T, Ballman KV (2013). Discovery and validation of a prostate cancer genomic classifier that predicts early metastasis following radical prostatectomy. PLoS One.

[74] Arrigoni E, Galimberti S, Petrini M, Danesi R, Di Paolo A (2016). ATP-binding cassette transmembrane transporters and their epigenetic control in cancer: an overview. Expert Opin Drug Metab Toxicol.

