# Supplementary material for: Prostate Tumor Overexpressed-1 (PTOV1) promotes docetaxel-resistance and survival of castration resistant prostate cancer cells
Source: Oncotarget. 2017 Jul 22;8(35):59165–80. doi: 10.18632/oncotarget.19467 (PMC5601723; doi:10.18632/oncotarget.19467)
Supplement: Supplementary file 1 [file oncotarget-08-59165-s001.pdf]

Prostate Tumor Overexpressed-1 (PTOV1) promotes docetaxel-resistance and survival of castration resistant prostate cancer cells*

SUPPLEMENTARY MATERIALS

Cell culture and reagents

Wnt3a conditioned medium was kindly provided by D. Arango (Vall d'Hebron Institut of Research, Barcelona). Briefly, mouse L1-Wnt3a (ATCC® CRL2647™) cells were cultured in DMEM supplemented with 10% FBS and 0.4 mg/ml G418. After 4 days, medium was collected, filtered and store at 4ºC. Fresh medium was added to cells and cultured for another 3 days. Medium was collected, filtered and mixed with the first batch of medium. For long storage, Wnt3a-conditioned medium was stored in aliquots at -20ºC. To activate the Wnt signaling pathway, Du145 cells were cultured for 24 h with Wnt3a-conditioned medium diluted 1:1 with supplemented RPMI medium. Where indicated, cells were treated for 24 h with 30 µM JNK inhibitor II (Calbiochem) or 60 µM iCRT14 (Tocris Bioscience).

Polysomal profiling and fraction analysis

Polysome profiles were analyzed as previously described [5]. Briefly, cells were cultured to 80% confluence, incubated for 5 min at 37 ºC with 100 µg/ml of cycloheximide (Applichem, Darmstadt, Germany). Twenty million cells were collected per condition, washed in PBS and lysated in polysome buffer (PB) containing 10 mM TRIS-HCL pH 7.4, 100 mM KCL, 10 mM $MgCl_2$, 1% TRITON-X100, 2.5 U/ml Turbo DNAse (Ambion, Carlbad, CA), 2 mM Dithiothreitol, 10 U/ml RNAse inhibitor (Promega) and 100 µg/ml Cycloheximide (CHX) (Applichem). All buffers were prepared in DEPC-treated water. Equal amount of cytoplasmic extracts were loaded onto a linear 10-50 % sucrose gradient prepared in PB using an automated gradient maker (Gradient Master, Biocomp). Polysomes were separated by 2.5 h centrifugation at 37,000 rpm using a Beckmann SW41 rotor. Gradients loaded on a Density Gradient Fractionation System (Beckman Coulter) were monitored by following absorbance at 254 nm (Econo UV Monitor, BioRad, Hercules, CA) and 30 fractions of 400 µL were collected using a using a fraction collector (BioRad). Samples were immediately frozen in dry ice. RNA was extracted from each fraction and analyzed by RT-qPCR as described above.

Colony formation assay

Cells ($3x10^3$ cells/well) were seeded in 6 wells plates in triplicates. After 7 days, cells were fixed in 4% formaldehyde solution and stained with 0.5% crystal violet, photographed, and colonies were scored.

Immunofluorescence

Cells ($2x10^5$) were seeded in sterilized glass coverslips in 24 wells plates. The day after, cells were washed twice with phosphate-buffered saline (PBS) and fixed with 4% paraformaldehyde for 30 minutes. Cells were incubated for 30 min with blocking buffer consisting of 3% BSA-PBS and 0.1 % saponine to permeabilized cells. Cells were washed twice with PBS and incubated 1.5 h in a humidified dark chamber with 5 µg/ml of PTOV1 antibody prepared in blocking buffer. Cells were washed three times with PBS and incubated 1h in a humidified dark chamber with 4 µg/ml of fluorophore-conjugated secondary antibody Alexa Fluor 488 (Invitrogen). Negative control was prepared by adding only the primary antibody. Each coverslip was placed on the mounting medium ProLong Diamond Antifade Mountant with DAPI (ThermoFisher).

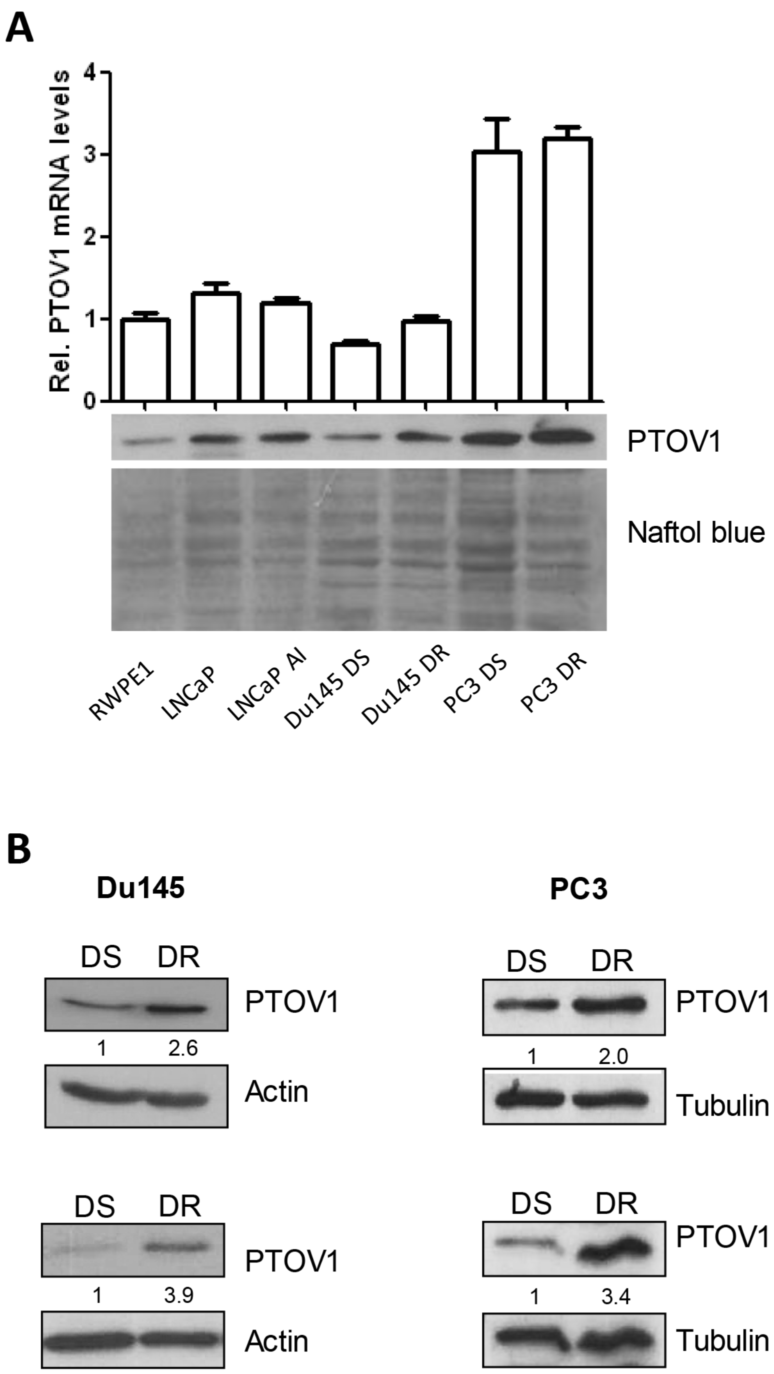

Supplementary Figure 1: PTOV1 expression levels in non-tumorigenic and tumorigenic prostate cells. **(A)** PTOV1 mRNA and protein levels are shown by qRT-PCR and Western blotting in normal prostate-derived cells (RWPE1), androgen sensitive cells (LNCaP), castration resistant LNCaP Androgen-Independent (AI), Du145 and PC3 cells, and CRPC cells resistant to docetaxel (DR). For normalization, the blot was stained with Naftol blue to show the proteins transferred to the membrane. **(B)** PTOV1 protein levels determined by Western blotting in two independent experiments in docetaxel sensitive (DS) and resistant (DR) Du145 and PC3 cells. The quantification of the signal is shown relative to the intensity of actin (or tubulin) signals.

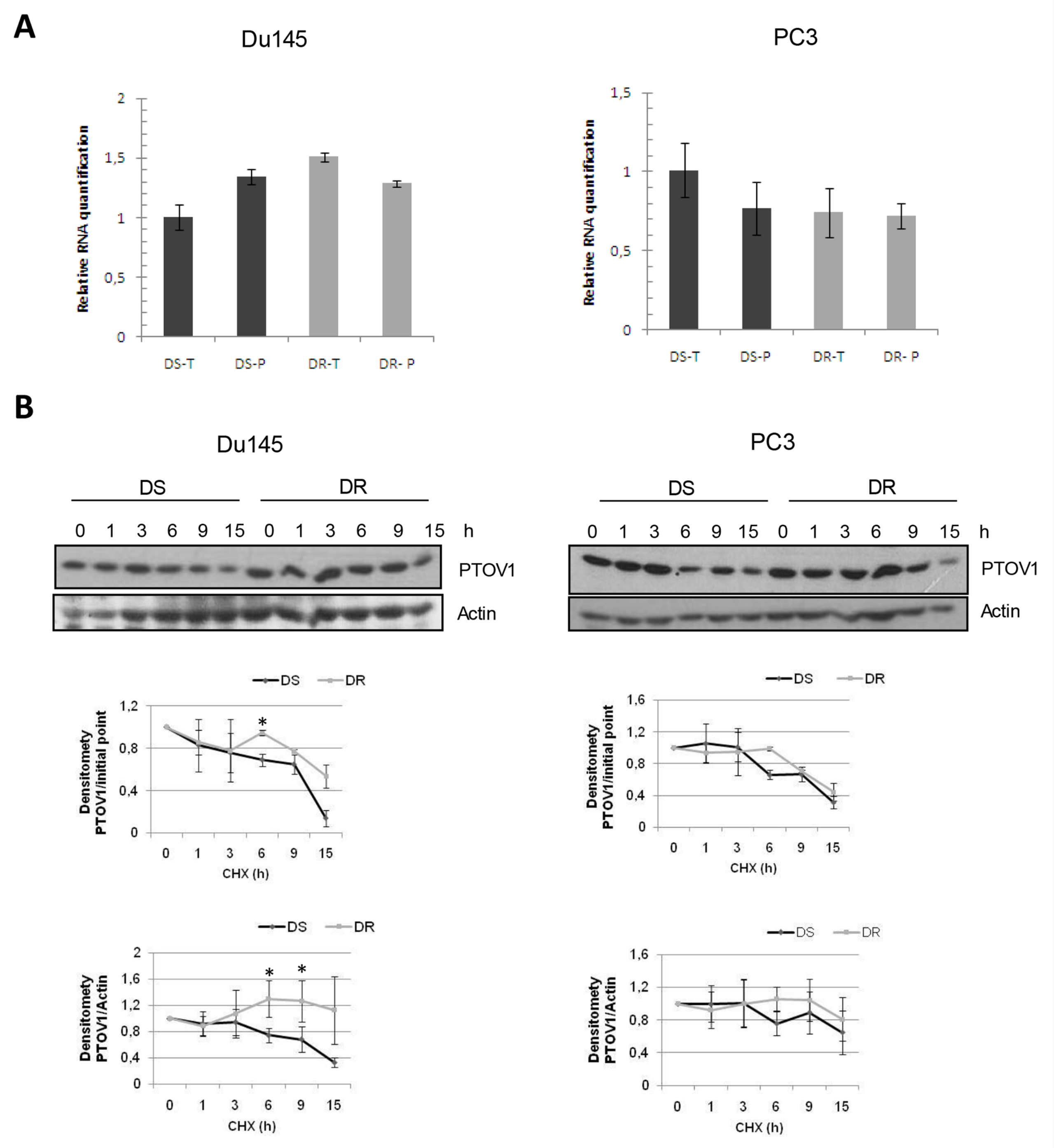

Supplementary Figure 2: Translational levels of PTOV1 in docetaxel resistant prostate cancer cells. (A) Representative histograms of *PTOV1* total RNA levels (t) and polysomal-bound mRNA (P) levels in DS and DR Du145 and PC3 cells. **(B)** Analysis of the stability of the protein PTOV1 in prostate cancer cells. Du145 and PC3 cells were treated with cycloheximide (CHX, 100 µg/ml) at the indicated time points. Upper panels show western blots, middle panels are the result of a scanning densitometry of PTOV1 signals related to initial controls (not treated cells), and bottom panels are from a scanning densitometry of PTOV1 signals related to actin. Densitometries were obtained by ImageJ software.

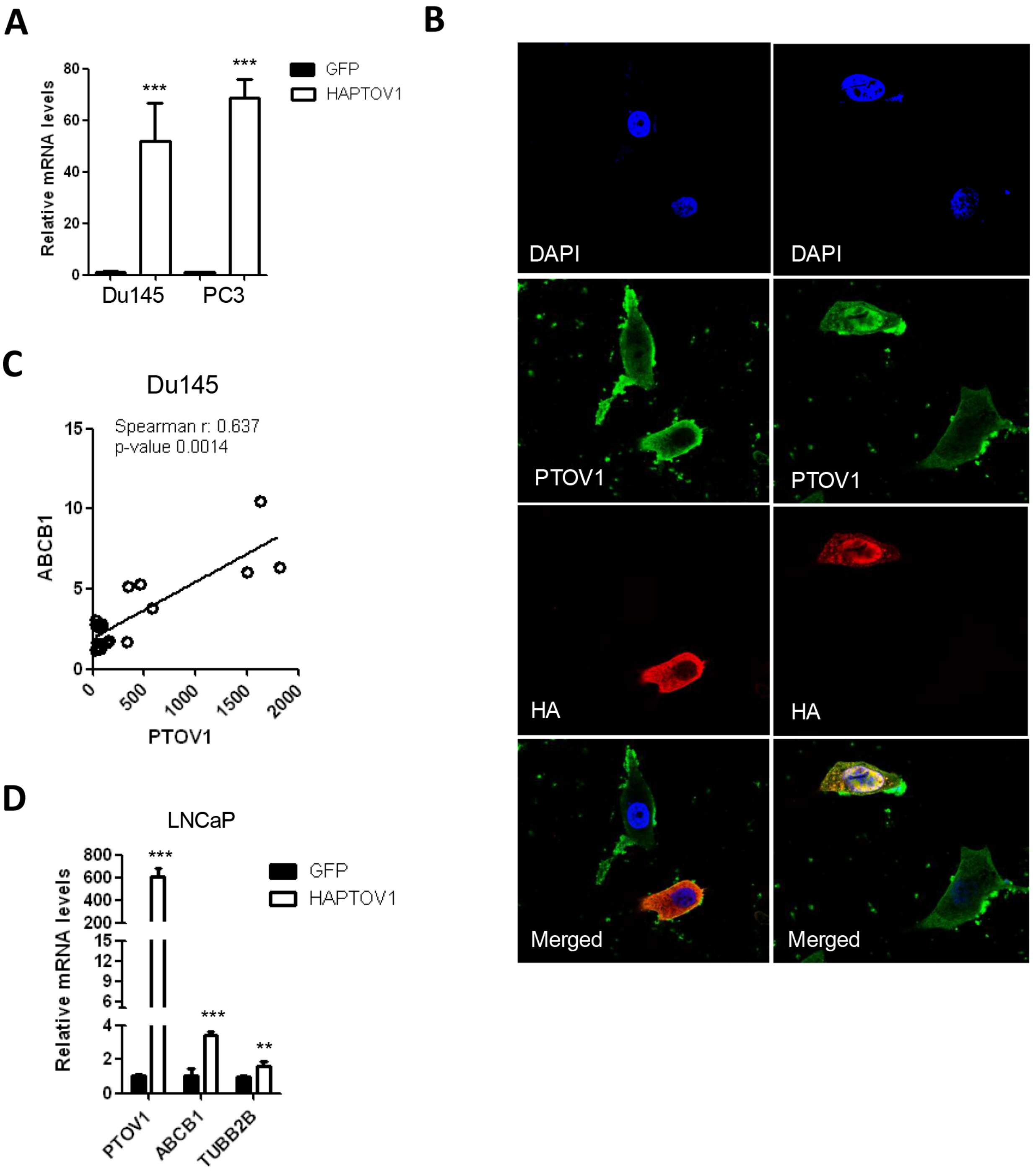

Supplementary Figure 3: The ectopic expression of PTOV1 in DS prostate cancer cells induces changes in the expression profiles. **(A)** The expression of *PTOV1* by qRT-PCR in DS DU145 and PC3 cells transduced with HAPTOV1 or a control lentivirus, as described in Figure 2 (mean ± S.D.). **(B)** Immunocytochemical co-localization of endogenous and ectopic (α-HA) PTOV1 in the cytoplasm and nucleus of PC3 cells transduced with HAPTOV1. **(C)** The histogram represents the distribution and correlation between *PTOV1* and *ABCB1* mRNA levels in Du145 cells expressing increased amounts of HAPTOV1 (3, 7.5, 15 and 30 µg). **(D)** The expression of *ABCB1* and *TUBB2B* genes by qRT-PCR in LNCaP cells transduced with HAPTOV1 or a control (GFP) (mean ± S.D.).

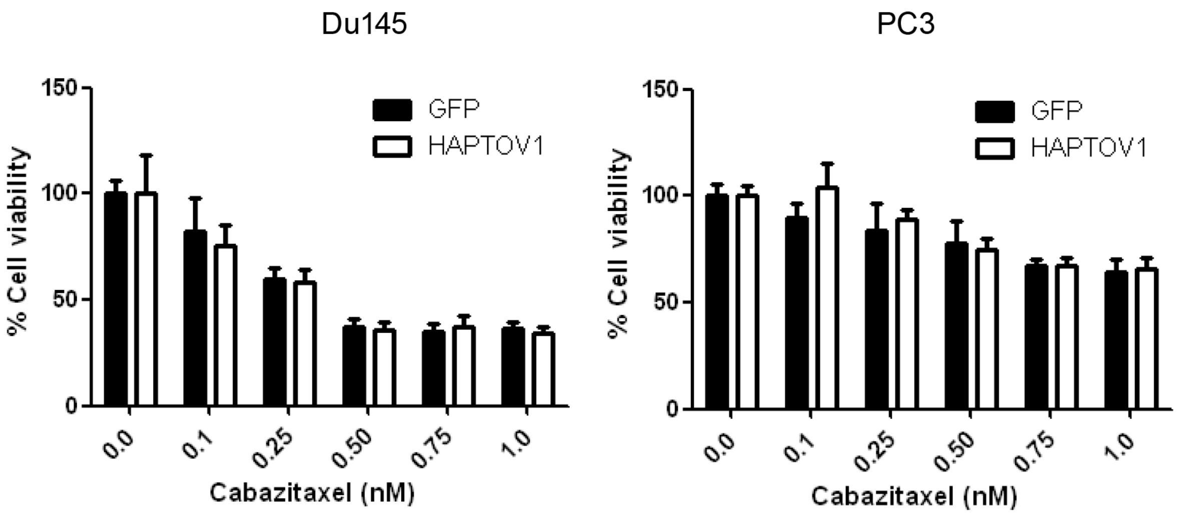

Supplementary Figure 4: PTOV1 does not promote resistance to cabazitaxel. DS-Du145 and DS-PC3 cells transduced with HAPTOV1 or a control (GFP) were treated with increasing doses of cabazitaxel for 48h. Cell viability was analyzed by crystal violet.

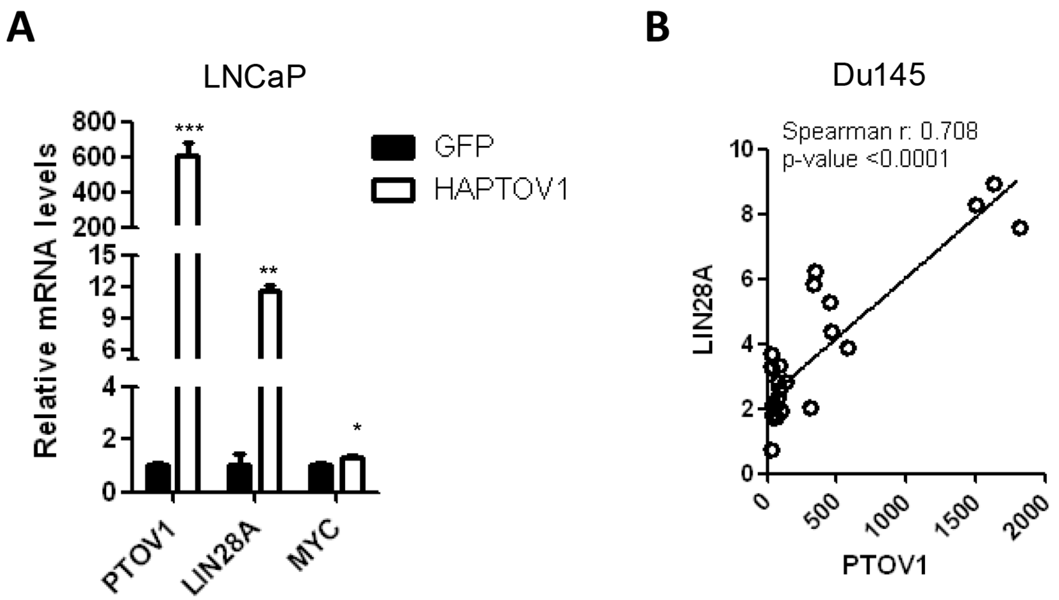

Supplementary Figure 5: PTOV1 induces the expression of self-renewal genes in prostate cancer cells. (A) Expression of *LIN28A* and *MYC* by qRT-PCR in LNCaP cells transduced with HAPTOV1 or a control lentivirus (GFP) (mean ± S.D.) **(B)** Histogram representing the distribution and correlation between *PTOV1* and *LIN28A* expression in Du145 cells expressing increased amounts of HAPTOV1.

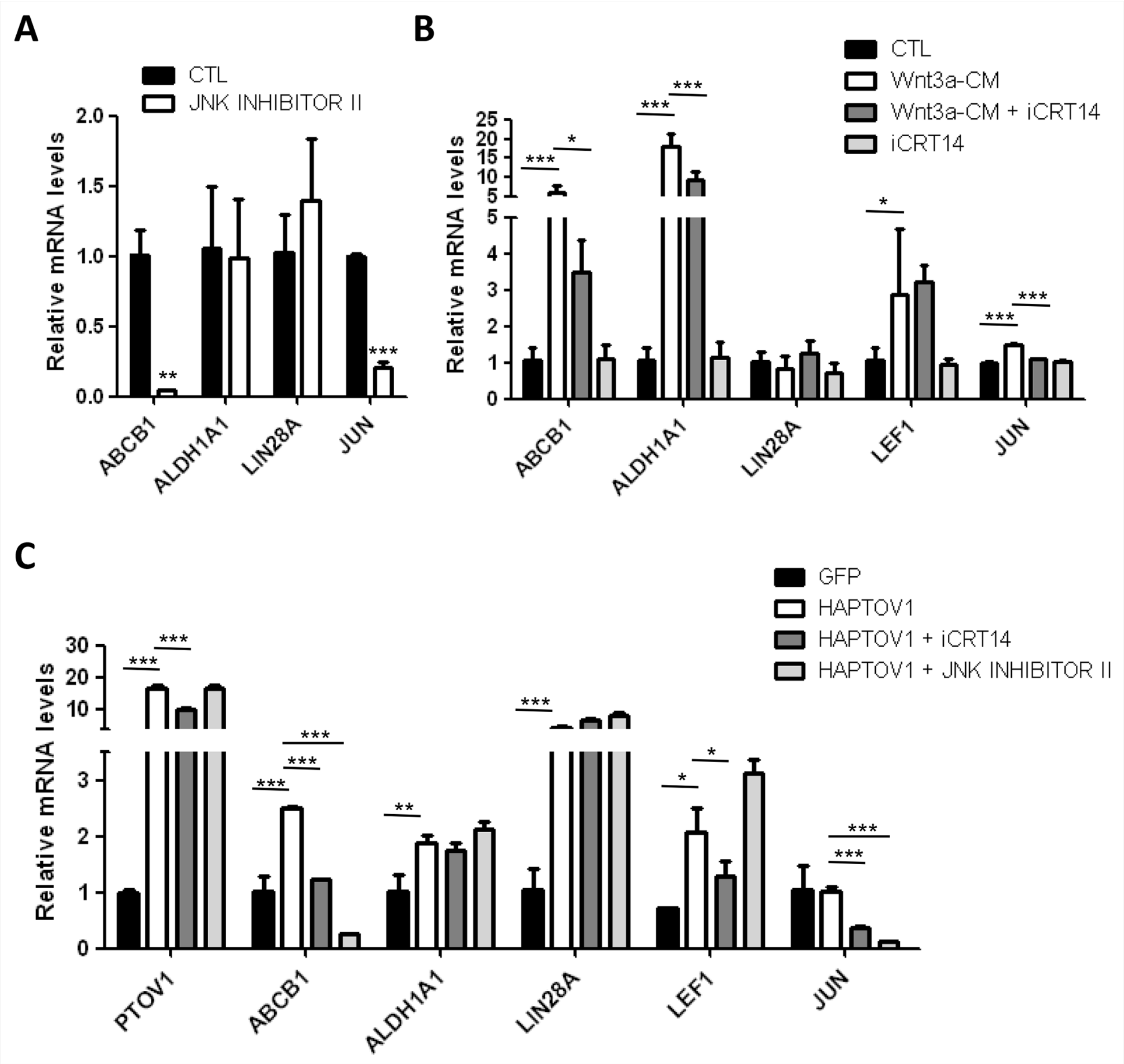

Supplementary Figure 6: The Jun and Wnt pathways are implicated in the transcriptional activity of PTOV1. (A) Gene expression was determined by qRT-PCR in DS-Du145 cells after 24 h of treatment with the JNK inhibitor II. **(B)** Gene expression in DS-Du145 cells analyzed after the addition of conditioned medium with Wnt3a (Wnt3a-CM), without and with a Wnt/β-catenin inhibitor (iCRT14), or control cells treated only with iCRT14. LEF1 expression levels indicate the activity of the Wnt pathway. **(C)** Gene expression was determined by qRT-PCR in DS-Du145 cells transfected with HAPTOV1 in the presence of the Wnt/β-catenin inhibitor (iCRT14) or JNK inhibitor II.

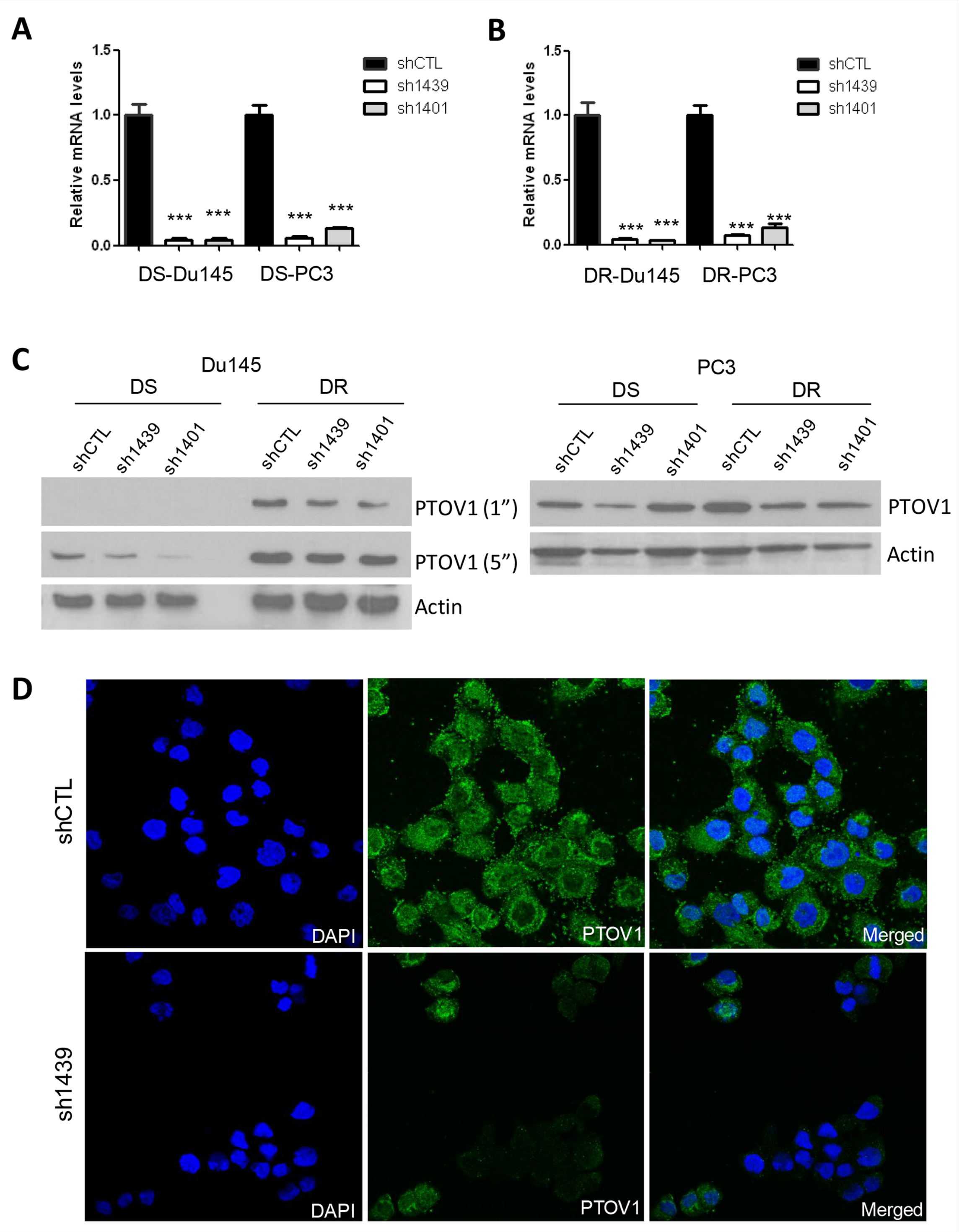

Supplementary Figure 7: The knockdown of PTOV1 in docetaxel sensitive (DS) and resistant (DR) PC cells efficiently decreases its endogenous gene expression. **(A)** The expression of PTOV1 in DS-Du145 and DS-PC3 cells knocked down by two different shRNA sequences (sh1439, sh1401) and a control shRNA (shCTL) was analyzed by qRT-PCR. **(B)** Shows the expression of PTOV1 by qRT-PCR in resistant DR-Du145 and DR-PC3 cells transduced with sh1439 and sh1401 lentivirus. **(C)** The expression of PTOV1 by Western blotting in DS and DR Du145 and PC3 cells. **(D)** Immunocytochemical localization of PTOV1 in DS-Du145 knockdown for PTOV1 by a shRNA sequence (sh1439).

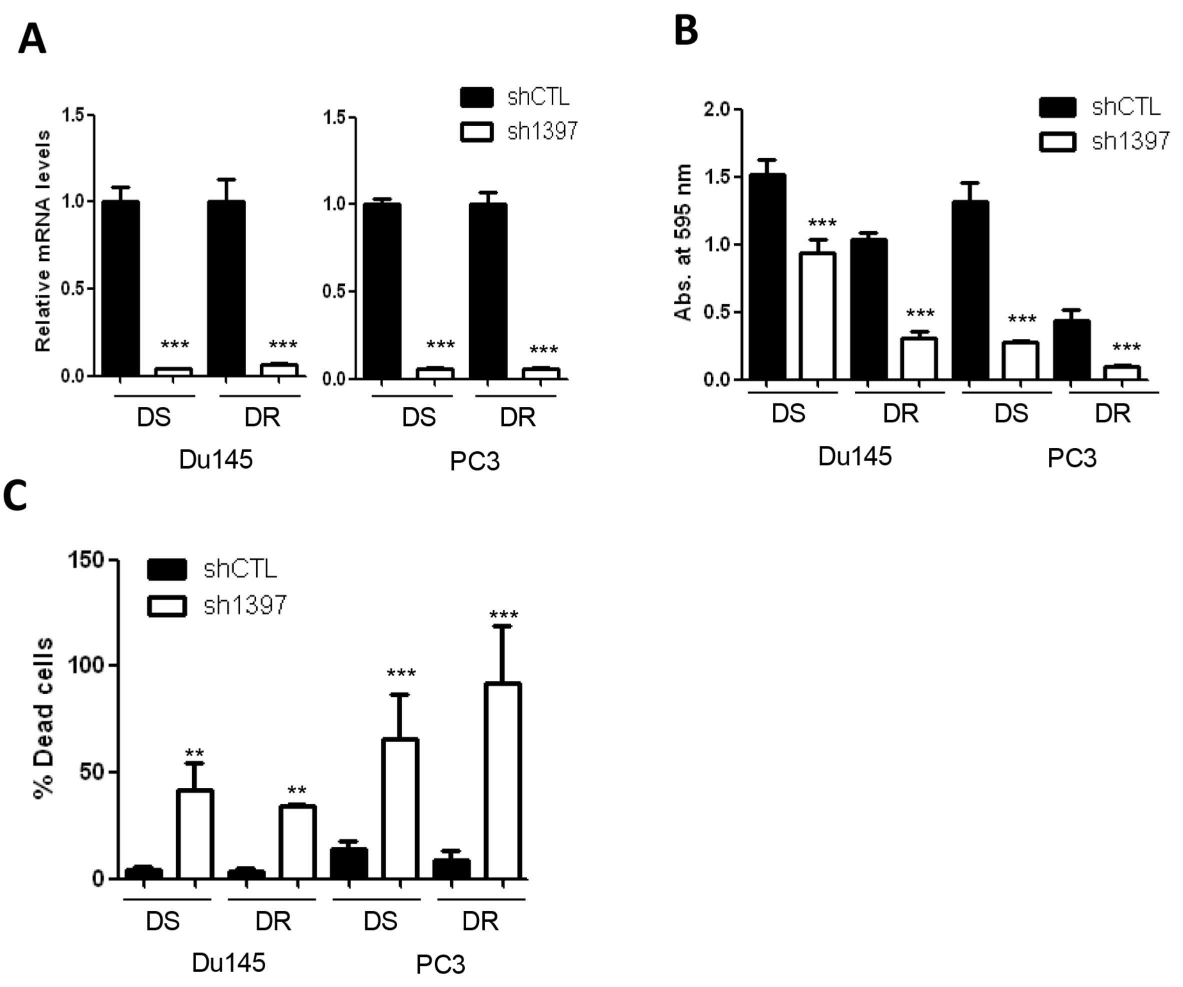

Supplementary Figure 8: The knockdown of PTOV1 in prostate cancer cells significantly decreases cell viability and increases apoptosis. (A) The expression of PTOV1 by qRT-PCR in DS and DR Du145 and PC3 cells transduced with sh1397 lentivirus compared to a control shRNA (shCTL). **(B)** Histogram representing the proliferation of DS and DR Du145 and PC3 cells knockdown for PTOV1 as in (A). Proliferating cells were determined by crystal violet staining and absorbance at 595 nm after 96 h from lentiviral transduction. **(C)** Histogram representing the number of apoptotic cells assessed by the Trypan blue exclusion in knockdown cells after 96 h from lentiviral transduction.

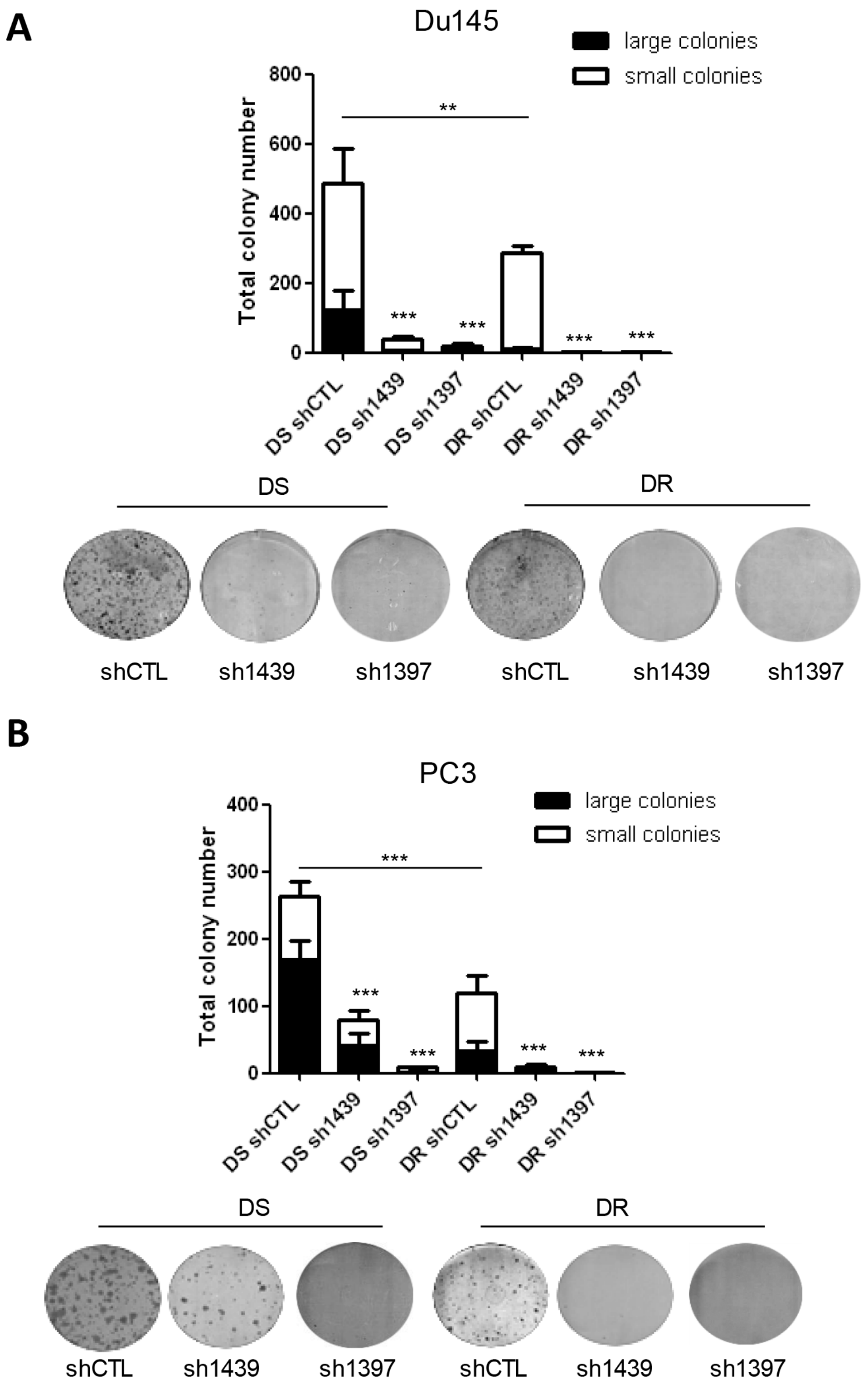

Supplementary Figure 9: Colony forming abilities of prostate cancer cells knockdown for PTOV1. Colonies formation abilities by DS and DR Du145 cells **(A)** and DS and DR PC3 cells **(B)** after the knockdown of PTOV1 (sh1439; sh1397), and a control shRNA (shCTL). After seven days, cells were stained with 0.5% crystal violet. Graphs show the quantification of the number of colonies, including large colonies (> 1mm Ø) and small colonies (< 1mm Ø). At the bottom, macroscopic images of the culture dishes are shown.

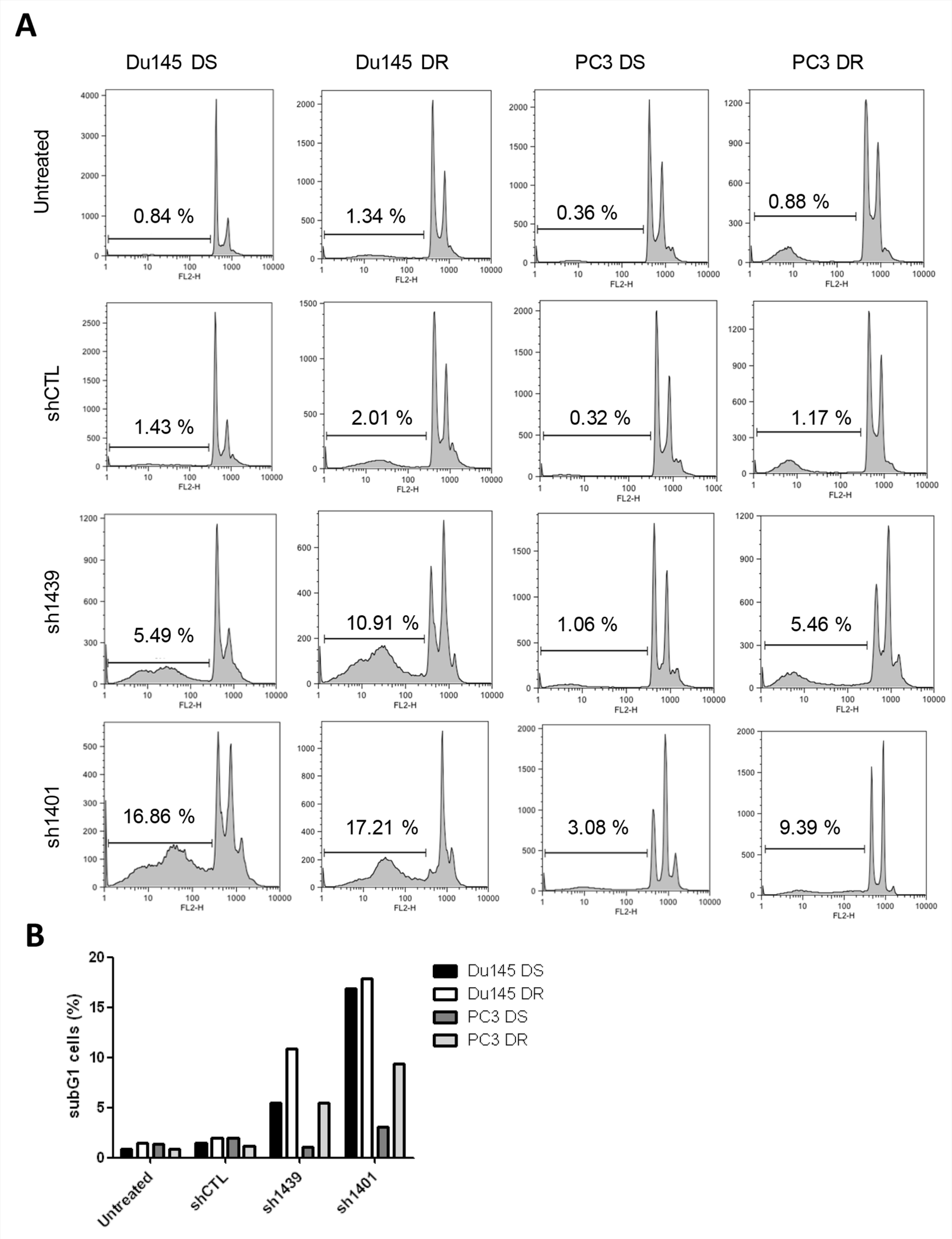

Supplementary Figure 10: Prostate cancer cells knockdown for PTOV1 accumulate in the sub-G1 peak of the cell cycle. **(A)** Histograms and **(B)** graph represent the cell cycle analysis by cytometry of total cells (viable and not viable cells) in DS and DR prostate cancer cells knockdown for PTOV1. Percentages shown in each histogram represent the proportion of cells in sub-G1 peak and reflect the proportion of dead cells.

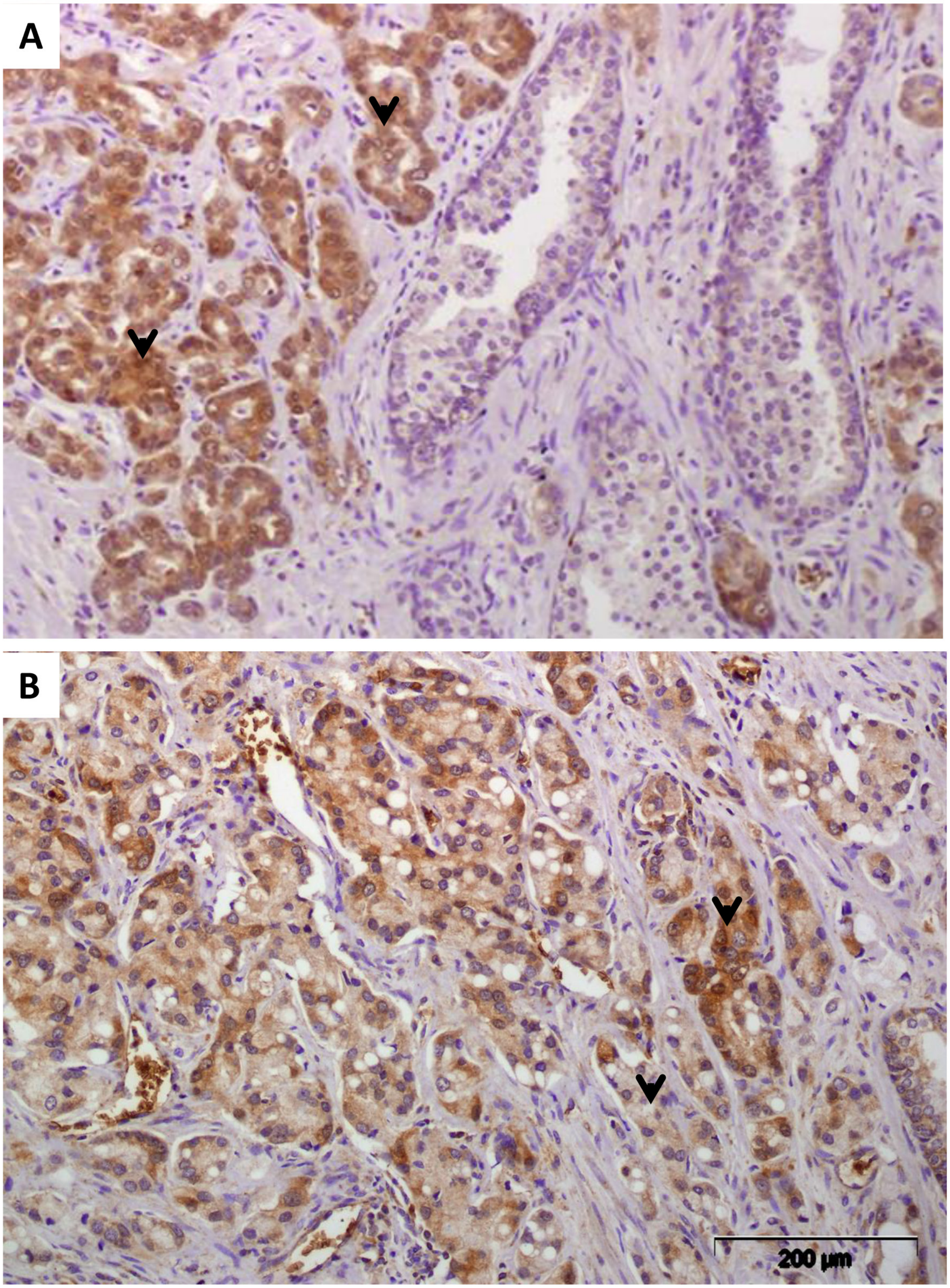

Supplementary Figure 11: Representative images from immunohistochemical staining of sections from two different human prostate tumors with specific antibodies to PTOV1. Slides were counterstained with hematoxylin. PTOV1 staining is low or undetectable in benign glands and strong expression is observed in cancer areas (arrow heads). Strong nuclear and cytoplasmic staining is observed in the majority of cancer cells in tumor **(A)**, however some tumor glands show a less intense staining, as in tumor **(B)**.

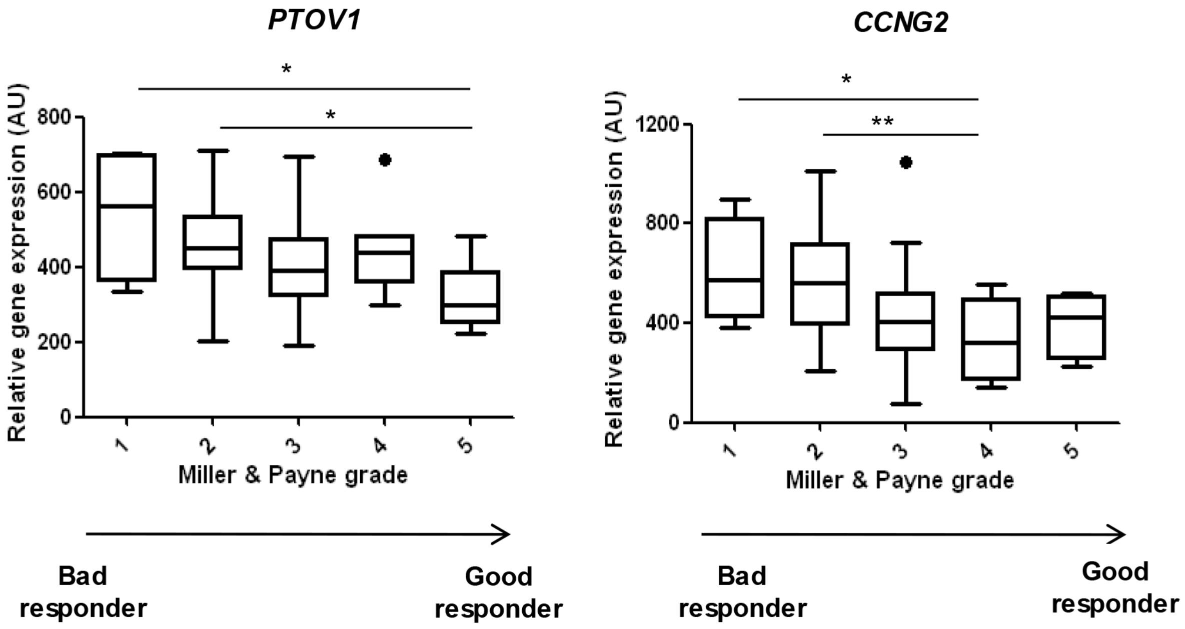

Supplementary Figure 12: The expression of *PTOV1* and *CCNG2* is significantly increased in breast tumors of patients with lower Miller and Payne grade (the group of bad responders to chemotherapy). Box and whisker plots representing *PTOV1* and *CCNG2* expression was obtained from published breast cancer gene expression profiles (GSE28844; grade 1 n=4; grade 2 n=24; grade 3 n=20; grade 4 n=7; grade 5 n=6). Patients with breast cancer were given neoadjuvant chemotherapy treatment based on anthracyclines and taxanes, and tumor samples were obtained before and after chemotherapy.

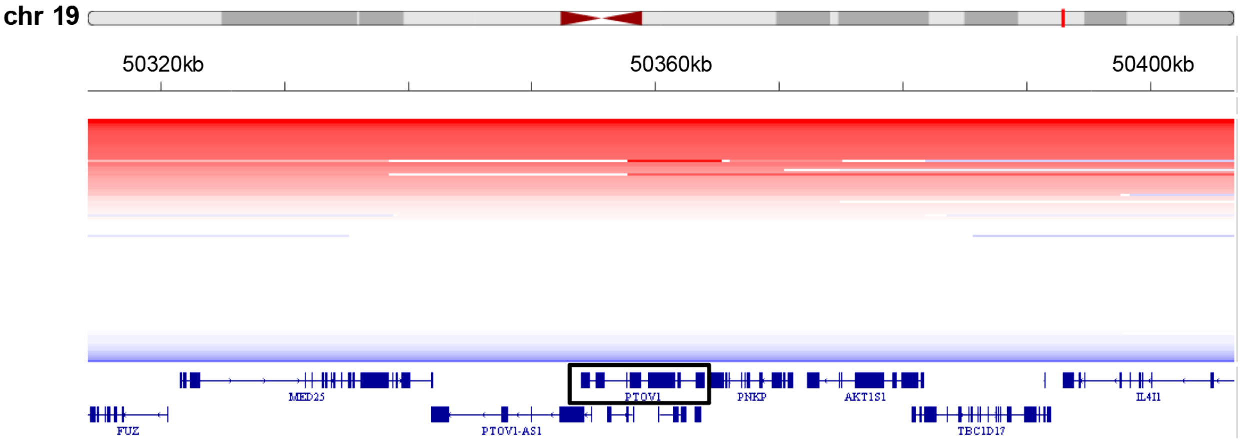

Supplementary Figure 13: Copy number status of PTOV1 locus. Color intensity and location are indicative of level and focality of amplification.

Supplementary Table 1: Primers used for real time RT-PCR transcript quantification

Gen	UPL probe	Forward primer 5’ > 3’	Reverse primer 5’ > 3’
ABCB1	#17	TGTGGGAAGAAGAGCACAGTGG	TTTATTTCTTTGCCATCAAGCA
CCNG2	#55	GGGGGTTGTTTTGATGAAAGT	TTGATCACTGGGAGGAGAGC
HMBS	#26	TGTGGTGGGAACCAGCTC	TGTTGAGGTTTCCCCGAAT
MYC	#34	CACCAGCAGCGACTCTGA	GATCCAGACTCTGACCTTTTGC
NANOG	#69	ATGCCTCACACGGAGACTGT	AGGGCTGTCCTGAATAAGCA
PTOV1	#9	GCTTCGTCAGTGCCATCC	TGAGTTGACACCACCAGGTC
TUBB2B	#78	TTGACCCCACTGGCAGTTAC	GATGGGCCCGAGGAACATA
β-ACTIN	#11	ATTGGCAATGAGCGGTTC	CGTGGATGCCACAGGACT
LEF1	#31	TGAATCAGGTACAGGTCCAA	CGTTGGGAATGAGCTTCG
JUN	#19	CCAAAGGATAGTGCGATGTTT	CTGTCCCTCTCCACTGCAAC
POU5F1	#52	GTGCCTGCCCTTCTAGGAAT	GGCACAAACTCCAGGTTTTCT
ALDH1A1	#34	GCTCTCCACGTGGCATCT	GCCCCATAACCAGGAACAAT
TUBB4A	#55	GGGGCAGTGACCTGCAA	GGGGACATAATTTCCTCCTGT
LIN28A	#23	GAAGCGCAGATCAAAAGGAG	GCTGATGCTCTGGCAGAAGT
